# Contribution of the TRPM4 Channel to Osteogenic Differentiation of Human Aortic Valve Interstitial Cells

**DOI:** 10.1161/JAHA.124.038542

**Published:** 2025-04-07

**Authors:** Margaux Aize, Arthur Boilève, Benoit D. Roussel, Laura Brard, Harlyne Mpweme Bangando, Corentin Kerevel, Alexandre Lebrun, Hind Messaoudi, Vladimir Saplacan, Alain Manrique, Romain Guinamard, Christophe Simard, the STOP‐AS Investigators

**Affiliations:** ^1^ Normandy University, UNICAEN, UR4650, Physiopathologie et Stratégies d’Imagerie du Remodelage cardiovasculaire (PSIR) Caen France; ^2^ Physiopathology and Imaging of Neurological Disorders (PhIND) Normandy University, UNICAEN, INSERM Caen France; ^3^ Univ Rouen Normandie, INSERM, ENVI UMR1096 Rouen France; ^4^ Department of Cardiovascular Surgery CHU de Caen Normandie Caen France

**Keywords:** aortic stenosis, TRPM4, valvular interstitial cells, Ion Channels/Membrane Transport

## Abstract

**Background:**

Aortic stenosis due to deleterious remodeling of the aortic valve is a health concern since it can be corrected only by valve replacement due to the poor knowledge of cellular mechanisms involved. Fibroblastic valvular interstitial cells (VICs) play a central role in valve leaflet stiffness by trans‐differentiation into osteoblast‐like cells leading to calcification. The TRPM4 (transient receptor potential melastatin 4) cation channel was shown to participate in cardiac fibroblast remodeling. It is also involved in radiation‐induced aortic valve remodeling in vivo in mice. We hypothesized that TRPM4 might participate in human VIC transition to osteoblastic phenotype.

**Methods:**

Human aortic valves were collected from patients undergoing surgical valve replacement. Isolated VICs were maintained 14 days in culture in standard or pro‐calcifying media and submitted to the TRPM4 inhibitor 9‐phenanthrol, or small hairpin RNA–TRPM4. Osteogenic differentiation was evaluated by measuring hydroxyapatite crystals by Alizarin red staining and protein expression of osteogenic markers.

**Results:**

Western blot on VICs revealed TRPM4 protein expression and channel functionality was confirmed by patch‐clamp recordings exhibiting a cationic current sensitive to voltage and internal Ca^2+^. VICs maintained in pro‐calcifying media exhibited a higher mineralization than in standard media, with an increase in osteogenic markers. Mineralization and osteogenic markers (bone morphogenetic protein 2, runt‐related transcription factor 2) were decreased when pro‐calcifying media contained 9‐phenanthrol or small hairpin RNA–TRPM4. Similarly, SMAD1/5 and nuclear factor of activated T‐cell pathways were stimulated in pro‐calcifying media conditions compared with standard media but reduced by 9‐phenanthrol or small hairpin RNA–TRPM4.

**Conclusions:**

TRPM4 participates in osteogenic differentiation of human VICs and thus appears as a target to prevent aortic valve remodeling.

## Graphical abstract

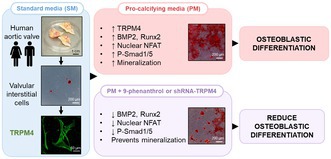

**Nonstandard Abbreviations and Acronyms**
9 PHE: 9‐phenanthrol
BMP2: bone morphogenetic protein 2
hVIC: human valvular interstitial cell
NFAT: nuclear factor of activated T cells
PM: pro‐calcifying media
Runx2: runt‐related transcription factor 2
shRNA: small hairpin RNA
SM: standard media
αSMA: α‐smooth muscle actin
TRPM4: transient receptor potential melastatin 4
VIC: valvular interstitial cell

**RESEARCH PERSPECTIVE**

**What Is New?**

- This study uncovers the contribution of the nonselective cation channel transient receptor potential melastatin 4 in the osteogenic remodeling of interstitial cells from human aortic valves (hVICs).
- Pharmacological inhibition as well as decreased expression of transient receptor potential melastatin 4 prevented in vitro expression of osteogenic markers and deposit of calcium salts, a key contributor to aortic stenosis.

**What Question Should Be Addressed Next?**

- Cellular signaling by which transient receptor potential melastatin 4 modulates expression of osteogenic markers remains to be determined as well as the role of transient receptor potential melastatin 4 regarding communication with other cell types involved in valve remodeling (eg, endothelial cells, immune cells).

Aortic stenosis is the most common valvular disorder. Its occurrence increases with aging (3% among the population aged >65 years and 4.6% among those aged >75 years),^1^, ^2^ predicting a future rise with the enhancement of life expectancy. It mainly results from the remodeling of the aortic valve leaflets with the development of fibrosis and, later, mineralization. It is difficult to correct other than by valve replacement, and suffers from the poor knowledge of the molecular actors involved. Valve mineralization is characterized by a deposition of hydroxyapatite crystals on the leaflets, leading to stiffness.^3^ Aortic valves are composed of a monolayer of valvular endothelial cells surrounding a tissue composed of fibroblastic valvular interstitial cells (VICs). In aortic stenosis, VICs differentiate into myofibroblasts expressing α‐smooth muscle actin (αSMA) and synthesizing collagen, which is responsible for fibrosis. Later, they differentiate into osteoblast‐like VICs responsible for calcification.^4^, ^5^ In this case, VICs express osteogenic proteins, such as the early marker BMP2 (bone morphogenetic protein 2).^6^ BMP2 is secreted and links to the BMP receptor types I and II at the surface of VICs,^7^ which in turn phosphorylates the SMAD1/5/8.^8^ This complex then links with SMAD4 and translocates in the nucleus to regulate expression of osteogenic markers such as Runx2 (runt‐related transcription factor 2), leading to VIC osteogenic activity responsible for valve mineralization.^9^ Ca^2+^ also plays a central role in VICs by mediating intracellular signals controlling gene expression and differentiation, in part by activating the nuclear factor of activated T cells (NFAT) pathway.^10^, ^11^ Ca^2+^ can link to calcineurin, a phosphatase that dephosphorylates NFAT, inducing the exposition of the nuclear domain. This factor acts as a transcriptional factor that can promote the expression of osteogenic markers.^11^ Modification of Ca^2+^ homeostasis was observed during valvular calcification.^12^ Therefore, molecules involved in Ca^2+^ homeostasis, including ion channels,^13^ might be valuable targets to modulate aortic stenosis.

The Ca^2+^‐activated nonselective monovalent cation channel TRPM4 (transient receptor potential melastatin 4)^14^ is involved in the remodeling of aortic valve induced by irradiation in mice.^15^ Indeed, wild‐type mice developed aortic stenosis associated with fibrosis 5 months after valve irradiation, which was less prominent in mice with *Trpm4* gene disruption (*Trpm4* ^*−/−*^). The cell type in which TRPM4 participates in valve remodeling is unknown, but it was shown that TRPM4 is involved in the transdifferentiation of fibroblasts into myofibroblasts in human atrial fibroblasts in culture.^16^ In this model, pharmacological inhibition of TRPM4 with 9‐phenanthrol reduced this transdifferentiation. In addition, this transdifferentiation was less prominent in atrial fibroblasts from *Trpm4* ^*−/−*^ compared with wild‐type mice.^16^ Regarding the relation of TRPM4 to aortic stenosis in human, it was observed an enhanced TRPM4 protein expression in calcified compared with noncalcified valves.^17^

TRPM4 is thus a candidate to participate in VIC osteogenic differentiation, leading to aortic stenosis. To test this hypothesis, we used human VICs (hVICs) in culture submitted to pro‐calcifying (PM) conditions inducing VIC mineralization by BMP2 and NFAT pathways activation. We observed that this phenomenon was reduced by TRPM4 pharmacological inhibition as well as gene repression using small hairpin RNA (shRNA).

## Methods

Original data used for western blot and polymerase chain reaction are included in the supplemental material. Other data sets generated and analyzed during the current study will be shared on reasonable request to the corresponding author.

### Patients

Human aortic valves were obtained anonymously from 81 patients (Tables S1 and S2) undergoing aortic valve replacement at Caen University Hospital Center. In agreement with French legislation, all procedures were carried out in accordance with *“ministère de l'enseignement supérieur, de la recherche et de l'innovation”* (DC‐2021‐4523) after informed consent of the patients and according to the principles outlined in the Declaration of Helsinki. Aortic stenosis was characterized by a maximal aortic jet velocity superior to 2 m.s^−1^, according to patient echocardiography parameters (Table S1).

### VIC Isolation

For most experiments, hVICs were obtained from calcified aortic valves (N=74). Note that for specific experiments indicated in the text, 7 noncalcified aortic valves were used. Leaflets were washed in 37 °C PBS and bathed into collagenase I solution (150 U.mL^−1^; Worthington Chemicals) diluted in DMEM, high glucose (4.5 g.L^−1^), GlutaMax (Gibco) for 10 minutes at 37 °C under gentle shaking to remove the endothelial layer. Samples were then placed in fresh collagenase I solution for 2 steps of 1 and 2 hours at 37 °C under high shaking. Cells were maintained in gelatin‐fibronectin (Sigma Aldrich) precoated flasks in standard media (SM) composed of DMEM, high glucose, and GlutaMax supplemented with 1% antibiotics (100 IU.mL^−1^ penicillin G‐Na, 50 IU.mL^−1^ streptomycin sulfate; Gibco), 10% FBS and 1% sodium pyruvate at 37 °C in 5% CO_2_ environment. Cells were used between passages 1 and 5.

### Reverse Transcriptase (Quantitative) Polymerase Chain Reaction

Precise protocol is described in Data S1 and primers are provided Table S3.

### Patch‐Clamp Recordings and Analysis

Single‐channel currents were recorded on hVICs using the inside‐out configuration of the patch‐clamp technique at room temperature (20–25 °C). An Axopatch 200B amplifier (Molecular Devices) and a Digidata 1322A A/D converter (Molecular Devices) were used to acquire and analyze the data in conjunction with pClamp software (Molecular Devices).

Bath solution contained, in mmol.L^−1^: 140 NaCl, 4.8 KCl, 1.2 MgCl_2_, 1 CaCl_2_, 10 HEPES, and 10 glucose (pH 7.4). Pipette solution contained, in mmol.L^−1^: 145 NaCl, 1.2 MgCl_2_, 1 CaCl_2_, 10 HEPES, and 10 glucose (pH 7.4). Standard internal solution contained, in mmol.L^−1^: 145 NaCl, 1.2 MgCl_2_, 1 CaCl_2_, 10 HEPES, and 10 glucose (pH 7.2). To evaluate ionic selectivity, the internal solution was, in mmol.L^−1^: 42 NaCl, 1.2 MgCl_2_, 1 CaCl_2_, 206 sucrose, 10 HEPES, and 10 glucose (pH 7.2) or 145 KCl, 1.2 MgCl_2_, 1 CaCl_2_, 10 HEPES, and 10 glucose (pH 7.2).

### shRNA‐TRPM4 Generation

A pLKO.1 puromycin plasmid (addgene No. 8453) was digested by EcoRI and AgeI (New England Biolabs Nos. R0552S and R0101S). Two shRNA‐TRPM4s were designed: SH1: 5′‐AAGATCTTCAAGAAGAAGACC‐3′, SH2: 5′‐AACGGATCCAGCTGCAGTTTA‐3′ (Eurogentec). pLKO.1 puromycin plasmid and shRNA‐TRPM4 were inserted in TOP10 bacteria (One Shot TOP10 C404010). Colonies were sequenced to account for insertion (Eurofins). Relevant colonies were amplified and plasmids with transgene were purified (NucleoBond Xtrad Midi/Maxi 740410.50). The construct was mixed with packaging plasmids (pRSV‐REV, pCMVΔR8.92 and pMD2G‐VSVG) for insertion in HEK‐293T cells by the calcium chloride method. Forty‐eight hours after transfection, supernatants that contained viral particles were recovered and centrifuged to pellet the viral particles. These were resuspended in 1% BSA‐PBS and quantified by HIV‐1 P24 Antigen ELISA (ZeptoMetrix). hVICs were transduced by 1 ng/cm^2^ of viral particles. For long‐term culture, viral particles were added every 7 days. To evaluate the potential effect of cell transduction by shRNA, a shScramble (5′‐AATGCATCAACTGGAATAGCA‐3′), which does not recognize the TRPM4 mRNA sequence, was used.

### Immunofluorescence

At the time of labeling, cells were washed 3 times with PBS and fixed in 4% paraformaldehyde‐PBS for 15 minutes. Cells were washed again 3 times with PBS and saturated in 1% BSA‐PBS for 1 hour at room temperature. Then, hVICs were permeabilized and incubated in 0.25% Triton‐PBS with primary antibodies (see Table S4 for details) overnight at 4 °C. Primary antibodies were detected using secondary antibodies (see Table S4) for 90 minutes at room temperature. Samples were mounted with Fluoromount G with DAPI (00495952; Thermo Fisher Scientific). Images were acquired with confocal microscope Leica DMi8 x40 and with Leica LAS X software (Leica) and analyzed by ImageJ software.

### hVIC Mineralization

Mineralization of hVIC was induced by 3, 7, or 14 days’ exposure to PM corresponding to DMEM with CaCl_2_ adjusted to 2.7 mmol.L^−1^ and NaH₂PO₄ to 2.5 mmol.L^−1^. When specified, the TRPM4 inhibitor, 9‐phenanthrol (9 PHE) at 3.10^−6^ mol.L^−1^ or shRNA (SH1, SH2 or SH1+2: 1 ng/cm^2^) were added in the PM. 9 PHE was diluted in DMSO. Maximal concentration of DMSO in culture media was 0.03%, and such concentration was added in control condition (culture without 9 PHE). The medium was changed twice a week. After 3, 7, or 14 days of treatment, the cells were fixed with ethanol, before adding 40 mmol.L^−1^ Alizarin red S (Himedia). Alizarin red S was dissolved by 0.28 mol.L^−1^ hexadecylpyridinium chloride (Himedia), and mineral deposition was quantified by absorbance at 562 nm using a Spark multimode microplate reader (Tecan). For each treatment condition, experiments were made in duplicate, and the mean value was determined.

### Cell Viability and Cycle

Cell viability and cycle was evaluated by flow cytometry. The precise protocol is described in the Supplemental Methods.

### Western Blot

Cells were cultured in SM or PM and with 9 PHE or shRNA when specified. At 7 or 14 days, total proteins were extracted with lysis buffer (50 mmol.L^−1^ Tris pH 7.4, 100 mmol.L^−1^ NaCl, 50 mmol.L^−1^ LiCl, 5 mmol.L^−1^ EDTA, 0.5% Triton x100, 0.5% deoxycholate, 0.05% SDS) and cocktail of protease and phosphatase inhibitors (Sigma‐Aldrich P8340; and Thermo Scientific 78 428). The pellet was resuspended in lysis buffer after ultracentrifugation (75 minutes at 110 000*g*). Protein tissue were extracted from human calcified and noncalcified aortic valves by 2 cycles of TissueLyser (Retsch MM400) in presence of zirconium silicate beads in RIPA lysis buffer with cocktail of protease and phosphatase inhibitors. The mixture was incubated for 60 minutes at 4 °C under agitation and 2 other cycles of TissueLyser were done. Supernatant were collected, centrifuged (75 minutes at 110 000*g*), and the pellet were resuspended in lysis buffer. Proteins were quantified with Pierce BCA Protein Assay Kit (Thermo Scientific); 15 μg/lane were run on 4% to 20% polyacrylamide gels. After transfer, polyvinylidene fluoride membranes were saturated with 3% BSA in 0.1% Tween‐tris buffered saline and incubated overnight at 4 °C with primary antibodies (see Table S4). The membranes were incubated in secondary antibodies (see Table S4) for 90 minutes at room temperature, and bands were visualized with Clarity Western ECL Substrate (Bio‐Rad) using ChemiDoc MP Imaging System (Bio‐Rad). The expression levels were quantified using Image Lab (Bio‐Rad) and corrected by normalization against total protein expression and stain free.

### Statistical Analysis

Statistical analyses were performed using Prism software version 10.0.2 (GraphPad Software Inc.). The threshold for statistical significance was set to *P*<0.05; “n” refers to the number of isolated cells examined, and “N” refers to the number of dissociations. Normality was verified by the Shapiro–Wilk test. Differences between 2 groups were assessed using the *t* test. Differences among ≥3 groups were assessed using the parametric 1‐way ANOVA and nonparametric Friedman test with Dunn's multiple comparisons test or uncorrected Fisher's least significant difference test (see figure legends). For data comparing time‐dependent samples and culture conditions, 2‐way ANOVA with uncorrected Fisher's least significant difference test were used. Results are reported as mean±SEM. In all figures, statistical significance was indicated as follows: *P<0.05, **P<0.01, ***P<0.001, ****P<0.0001, ns=nonsignificant.

## Results

### TRPM4 mRNA and Protein Are Expressed on hVICs

**Figure 1:**
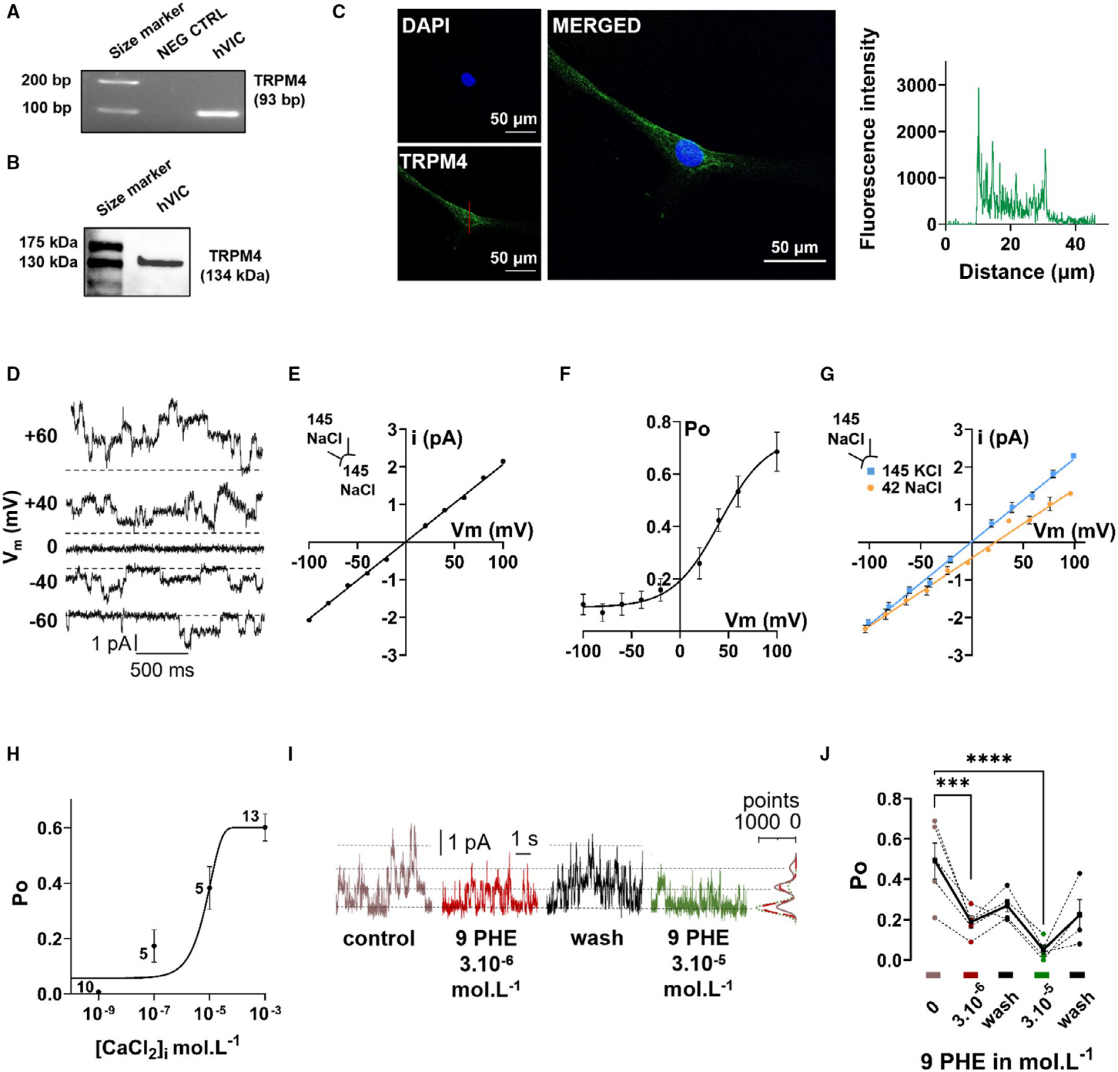
TRPM4 mRNA, protein expression and typical TRPM4 current in isolated hVICs. **A**, TRPM4 mRNA in hVICs detected by polymerase chain reaction. H_2_O was used as negative control. **B**, Representative western blot of TRPM4 protein in hVICs. **C**, Immunostaining of TRPM4 (green) on isolated hVICs. Nuclei were labeled with DAPI (blue). Scale bar=50 μm. Graphic in the right panel represents the profile of TRPM4 staining through the cell (according to the red vertical line in lower left image of TRPM4). **D**, Single‐channel tracings recorded at various membrane potentials (*V*_m_) from an inside‐out patch from hVIC. Pipette and bath contained 145 mmol.L^−1^ NaCl standard solution (CaCl_2_=10^−3^ mol.L^−1^). Dashed lines indicate current level when all channels are closed. At least 4 similar channels are present in the patch. **E**, Current voltage relationship (*i*/*V*_m_) under the same conditions as in **D**. Data points were fitted by linear regression indicating a conductance *g*=20.3±0.3 pS and a reversal potential=−0.11±0.40 mV (n=28, N=7). **F,** Voltage dependence of open probability (*P*_o_). In conditions described in **D**. *P*_o_ was determined at various *V*_m_ (n=12, N=6). Data were fitted to a Boltzmann equation. **G,** Current voltage relationship (*i*/*V*_m_) with the 145 mmol.L^−1^ NaCl standard solution in the pipette and the 145 mmol.L^−1^ KCl solution (blue squares; n=7, N=5) or 42 mmol.L^−1^ NaCl solution (orange circles; n=4, N=3) in the bath. Data points were fitted by linear regression. **H,** Values of *P*_o_ at various [Ca^2+^]_i_ (*V*_m_=+40 mV; symmetrical 145 mmol.L^−1^ NaCl standard solution). Numbers inside of data points indicate the number of experiments. Typical current traces are provided in the inset for the same patch at 10^−3^ or 10^−7^ mol.L^−1^ [Ca^2+^]_i_. **I**, Single‐channel current recorded from an inside‐out patch illustrating the blocking effect of 9 PHE applied at the inside of the membrane at 3.10^−6^ or 3.10^−5^ mol.L^−1^ (*V*_m_=+40 mV; symmetrical 145 mmol.L^−1^ NaCl standard solution). Corresponding amplitude histograms are provided on the right. Note that it indicates that 9 PHE decreased channel activity with no effect on single‐channel current amplitude. **J**, *P*_o_ determined in each condition for 5 experiments similar to that shown in **E**. Circles linked by dashed lines correspond to single experiments. Plain black squares linked by plain line correspond to the mean±SEM. Analyzed with mixed‐effects analysis (**J**) with uncorrected Fisher's least significant difference test. ***P<0.001, ****P<0.0001. 9 PHE indicates 9‐phenanthrol; hVIC, human valvular interstitial cell; NEG CTRL, negative control; *P*_o_, open probability; and TRPM4, transient receptor potential melastatin 4.

hVICs were isolated from aortic valves and maintained in SM. Their VIC phenotype was confirmed by quantitative polymerase chain reaction unmasking the expression of the VIC marker αSMA but not platelet endothelial cell adhesion molecule 1 and vascular endothelial‐cadherin known to be valvular endothelial cell markers (Figure S1). TRPM4 was searched for in hVICs in culture. mRNA TRPM4 transcript was detected (Figure 1A). In addition, when analyzing western blot, a band was detected close to a 130 kDa size marker consistent with the TRPM4 size (134 kDa) (Figure 1B). TRPM4 protein localization was assessed by confocal microscopy and mainly appeared at the plasma membrane (Figure 1C).

### Typical TRPM4 Current on hVIC

Single‐channel currents were recorded on hVICs in culture in SM in the inside‐out configuration of the patch‐clamp technique, first using symmetrical 145 mmol.L^−1^ NaCl solution on both side of the membrane (1 mmol.L^−1^ CaCl_2_). Unitary currents were detected (Figure 1D). The current/voltage relationship reveals a linear current with a slope conductance g=20.3±0.3 pS and a reversal potential=−0.1±0.4 mV (n=28, N=7) (Figure 1D and 1E). Open probability plotted as a function of voltage indicates a higher activity in positive compared with negative voltages (Figure 1F). Data fitted to a Boltzmann function indicate a maximal open probability of 0.77±0.06 and a voltage for half‐maximal activation *V*_0.5_=+34.2±6.1 mV (n=12, N=6). Reducing NaCl at the inside of the membrane to 42 mmol.L^−1^ shifted reversal potential to +24.2±1.9 mV (*g*=18.6±0.9 pS) (n=7, N=5) (Figure 1G), corresponding to a permeability ratio P_Na_/P_Cl_=8.96, according to the Goldman–Hodgkin–Katz equation. Selectivity among cations was assessed by using a 145 mmol.L^−1^ KCl solution at the inside of the membrane. Both *g*=21.6±0.4 pS and reversal potential=−0.6±0.4 mV were not significantly different than under symmetrical conditions and indicates a permeability ratio *P*_K_/*P*_Na_=1.02 (Figure.1G) (n=4, N=3). Channel sensitivity to internal Ca^2+^ was assessed by changing [Ca^2+^]_i_ from 10^−9^ to 10^−3^ mol.L^−1^ in the symmetrical NaCl 145 mmol.L^−1^ conditions at *V*_m_=+40 mV. It revealed a sigmoidal rise in open probability when [Ca^2+^]_i_ was increased with a concentration for half‐maximal activation EC_50_=2.4×10^−6^ mol.L^−1^ (Figure 1H). Channel sensitivity to internal applications of the TRPM4 inhibitor 9 PHE was assessed in the 145 mmol.L^−1^ NaCl conditions (V_m_=+40 mV). 9 PHE at 3.10^−6^ and 3.10^−5^ mol.L^−1^ significantly and reversibly reduced open probability to 30±4% and 13±6% that of control, respectively (n=12, N=5 and n=8, N=4, respectively) (Figure 1I–J). Altogether, it indicates that hVICs express a current with typical features of TRPM4 (single‐channel conductance, ion selectivity, voltage and Ca^2+^ sensitivity, pharmacology). It appeared on 79±1% of patches, when analyzed for each patient, with a mean of 3.0±0.6 channels per patch (n=130, N=18).

### 9‐Phenanthrol Prevents hVIC Mineralization

To evaluate the effect of TRPM4 on hVIC mineralization, cells were submitted to PM up to 14 days. The functional expression of TRPM4 after 14 days in PM was assessed by inside‐out patch‐clamp recordings in similar conditions as described for SM conditions. The ratio of patches with TRPM4 channels (33/33) was significantly higher than that observed for cells maintained in SM (31/130) according to the Fisher exact test (*P*=0.0008). Moreover, the mean number of TRPM4 channels per patch was significantly higher in the cells maintained in PM (5.4±0.5) (n=33, N=3) than in cells maintained in SM (*P*=0.003). Note that none of the biophysical properties (conductance, cation selectivity, dependence on voltage and internal calcium) were different between SM and PM treatments (Figure S2).

**Figure 2:**
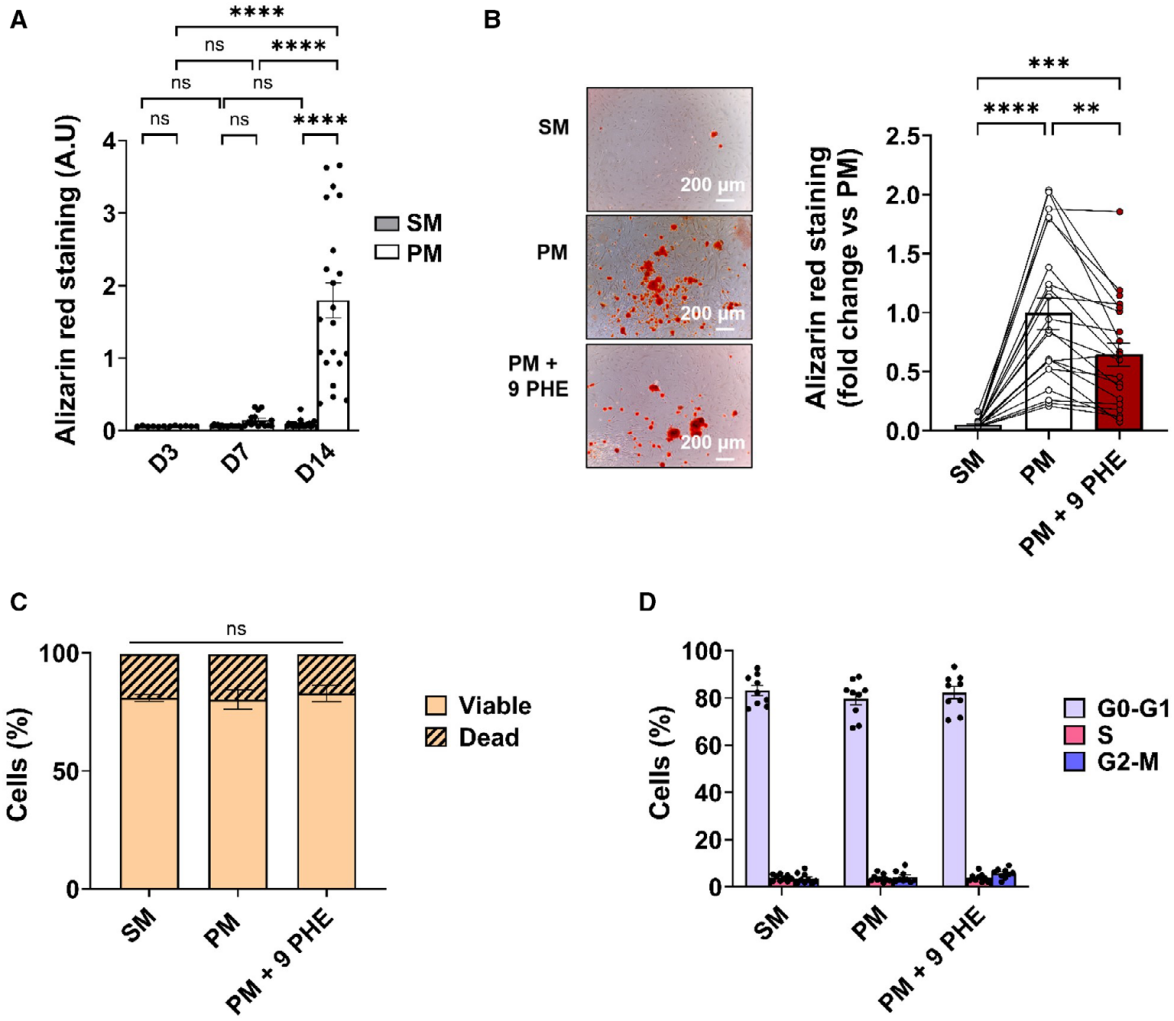
TRPM4 participates in hVIC mineralization. **A**, Calcium deposits measured by Alizarin red staining on hVICs. Cells were cultured in SM or PM media during 3, 7, or 14 days (D3, D7, and D14). Histograms are mean±SEM (N=6 for D3; N=15 for D7; N=21 for D14) in arbitrary units. **B**, Alizarin red staining of hVICs cultured in SM, PM, and PM+9 PHE 3.10^−6^ mol.L^−1^ (PM+9 PHE) for 14 days (N=21). Left panel shows representative pictures of the same culture in the 3 conditions. Right panel shows the fold change vs PM of Alizarin red quantification for each condition. Circles connected by lines represent data for each patient measured in the 3 conditions (average of duplicate). Histograms represent the mean±SEM (N=21). **C**, hVIC viability was evaluated by flow cytometry using propidium iodide experiment after 14 days of culture in SM, PM, or PM+9 PHE (N=11). **D**, hVIC cell cycle was determined by flow cytometry after 14 days of culture in SM, PM, or PM+9 PHE (N=9). Histograms are mean±SEM. Analyzed with 2‐way ANOVA (**A**) with uncorrected Fisher's least significant difference test and Friedman test (**B**) with Dunn's multiple comparisons test, with mixed‐effects analysis (**C**) with uncorrected Fisher's least significant difference test. *P<0.05, **P<0.01, ***P<0.001, ****P<0.0001, ns=nonsignificant. 9 PHE indicates 9‐phenanthrol; G0–G1, growth 0 and growth 1 phase; G2‐M, growth 2 phase and mitosis; hVIC, human valvular interstitial cell; PM, pro‐calcifying media; S, synthesis phase; SM, standard media; and TRPM4, transient receptor potential melastatin 4.

Mineralization was quantified by calcium deposit measurements using Alizarin red staining. No significant differences in Alizarin red staining between SM and PM conditions were detected at 3 and 7 days of treatment but a significant increase appeared at 14 days (Figure 2A). Note that in SM condition, no variation of staining was detected among time. Application of 9 PHE (3.10^−6^ mol.L^−1^) during culture in PM condition significantly prevented the mineralization of 35% after 14 days of treatment (N=21) (Figure 2B). To evaluate the effect of 9 PHE treatment on cell viability, flow cytometry assays were performed at 14 days. Close to 80% of cells were viable without difference between culture conditions (SM versus PM; PM versus PM+9 PHE) (N=11) (Figure 2C). Cell cycle was evaluated by flow cytometry at 14 days. Most of the cells were in the G0 to G1 phase without difference between culture conditions (N=9) (Figure 2D). Thus, the effect of 9 PHE on hVIC mineralization was not due to modifications of cell viability and cycle.

### 9 PHE Downregulates Osteogenic Markers

**Figure 3:**
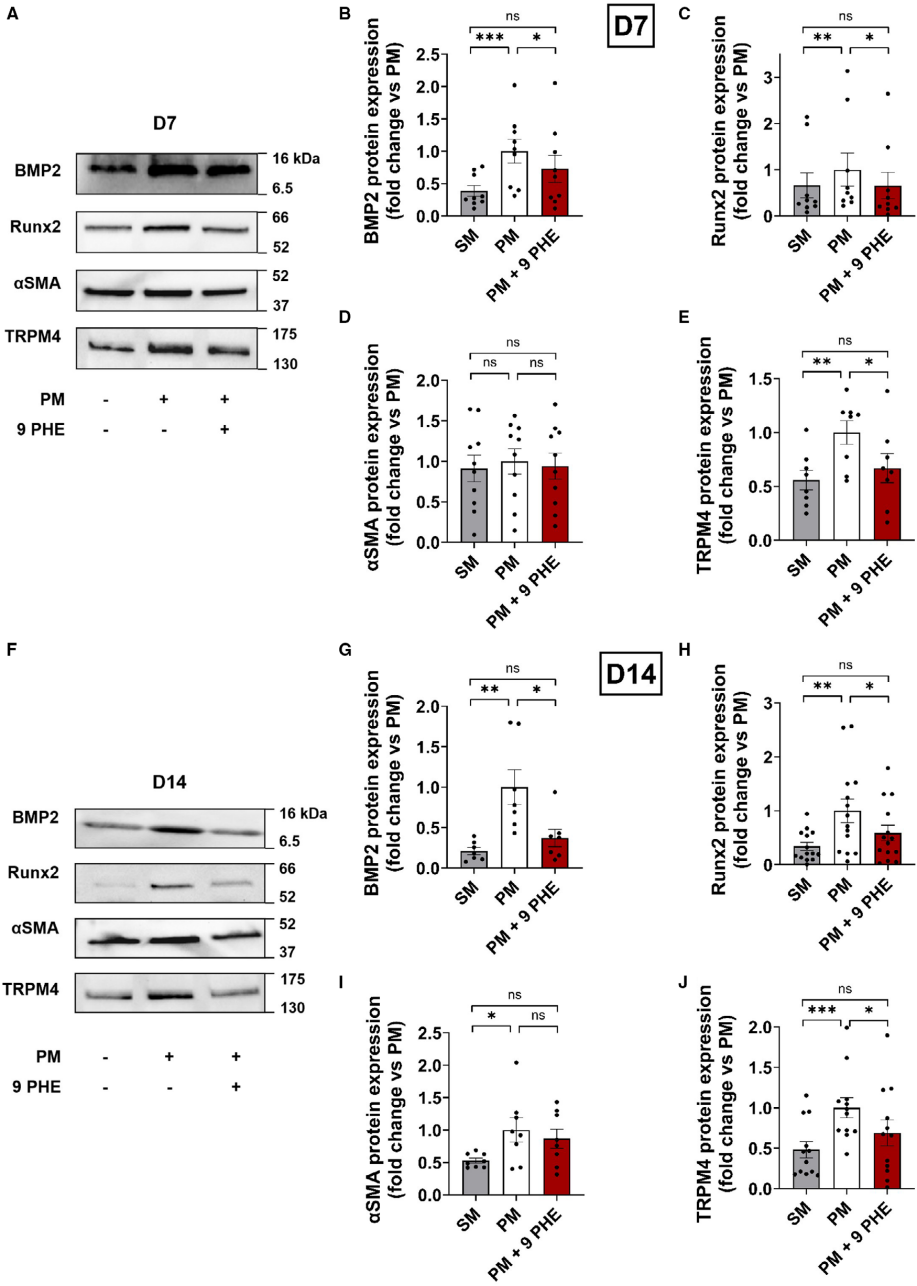
Reduction of osteogenic markers under pharmacological inhibition of TRPM4. **A** and **F**, Representative western blots of BMP2, Runx2, αSMA, and TRPM4 protein expression in hVICs after 7 (**A**) and 14 days (**F**) of culture. As indicated under panels, cells were cultured in SM conditions or in PM conditions without or with 9 PHE 3.10^−6^ mol.L^−1^. **B**–**E** and **G**–**J**, Quantification of intensity labeling of BMP2 (N=9 and 7 for **B** and **G**); Runx2 (N=9 and 14 for **C** and **H**); αSMA (N=10 and 8 for **D** and **I**) and TRPM4 (N=8 and 12 for **E** and **J**) after 7 days (**B–E**) and 14 days (**G–J**) of hVIC maintained in SM, PM, and PM+9 PHE culture conditions. Histograms are mean±SEM of fold change vs PM. Analyzed with Friedman (**B**, **C**, **G**, **H**, **J**) with Dunn's multiple comparisons and 1‐way ANOVA (**D**, **E**, **I**) with uncorrected Fisher's least significant difference multiple comparisons test. *P<0.05, **P<0.01, ***P<0.001, ns=nonsignificant. 9 PHE indicates 9‐phenanthrol; BMP2, bone morphogenetic protein 2; hVIC, human valvular interstitial cell; PM, pro‐calcifying media; Runx2, runt‐related transcription factor 2; SM, standard media; αSMA, α‐smooth muscle actin; and TRPM4, transient receptor potential melastatin 4.

Osteogenic markers (BMP2 and Runx2) as well as αSMA and TRPM4 were assessed by western blot in SM, PM, or PM+9 PHE 3.10^−6^ mol.L^−1^ conditions. At 7 days, BMP2 and Runx2 expression were increased in PM conditions compared with SM by 156% (N=9) and 52% (N=9), respectively. These variations were partly prevented by 9 PHE treatment (Figure 3A through 3C). At 14 days, BMP2 expression was increased by 400% (N=7) and Runx2 by 250% (N=14) in PM compared with SM conditions (Figure 3F through 3H). These increases were partially prevented by 9 PHE. Expression of the myofibroblast marker αSMA was similar in all conditions at 7 days (N=10) but was increased by 89% (N=8) in PM conditions at 14 days (Figure 3D and 3I). Regarding TRPM4, its expression was significantly increased in PM condition compared with SM at 7 days and this increase was significantly reduced in the presence of 9 PHE (N=8) (Figure 3E). These variations were more pronounced at 14 days (N=12) (Figure 3J).

TRPM4 protein expression was evaluated by western blot on calcified and noncalcified valves. The measurements were made at the tissue and cell levels. TRPM4 protein expression was higher on calcified valves than in noncalcified ones, even if not reaching statistical significance (N=7 for each group; *P*=0.12). In addition, we performed western blot to evaluate the expression of TRPM4 on hVICs issued from noncalcified valves (N=10) and compare them to those from calcified ones (N=8). It appeared that TRPM4 expression was significantly higher in hVICs from calcified valves (Figure S3). These data indicate that the increase in TRPM4 expression that we induced in vitro by PM conditions leading to calcification is also present in vivo in case of valve calcification.

### Effect of TRPM4 Repression by shRNA on hVIC Mineralization

hVICs were transduced with 2 different shRNA‐TRPM4s (SH1, SH2 or SH1+2). The efficient range of shRNA was tested for several concentrations ranging from 1 to 5 ng/cm^2^ in SM (Figure S4); 1 ng/cm^2^ was considered appropriate regarding efficiency for protein reduction and avoidance of potential cellular damage. It was thus used for following experiments.

**Figure 4:**
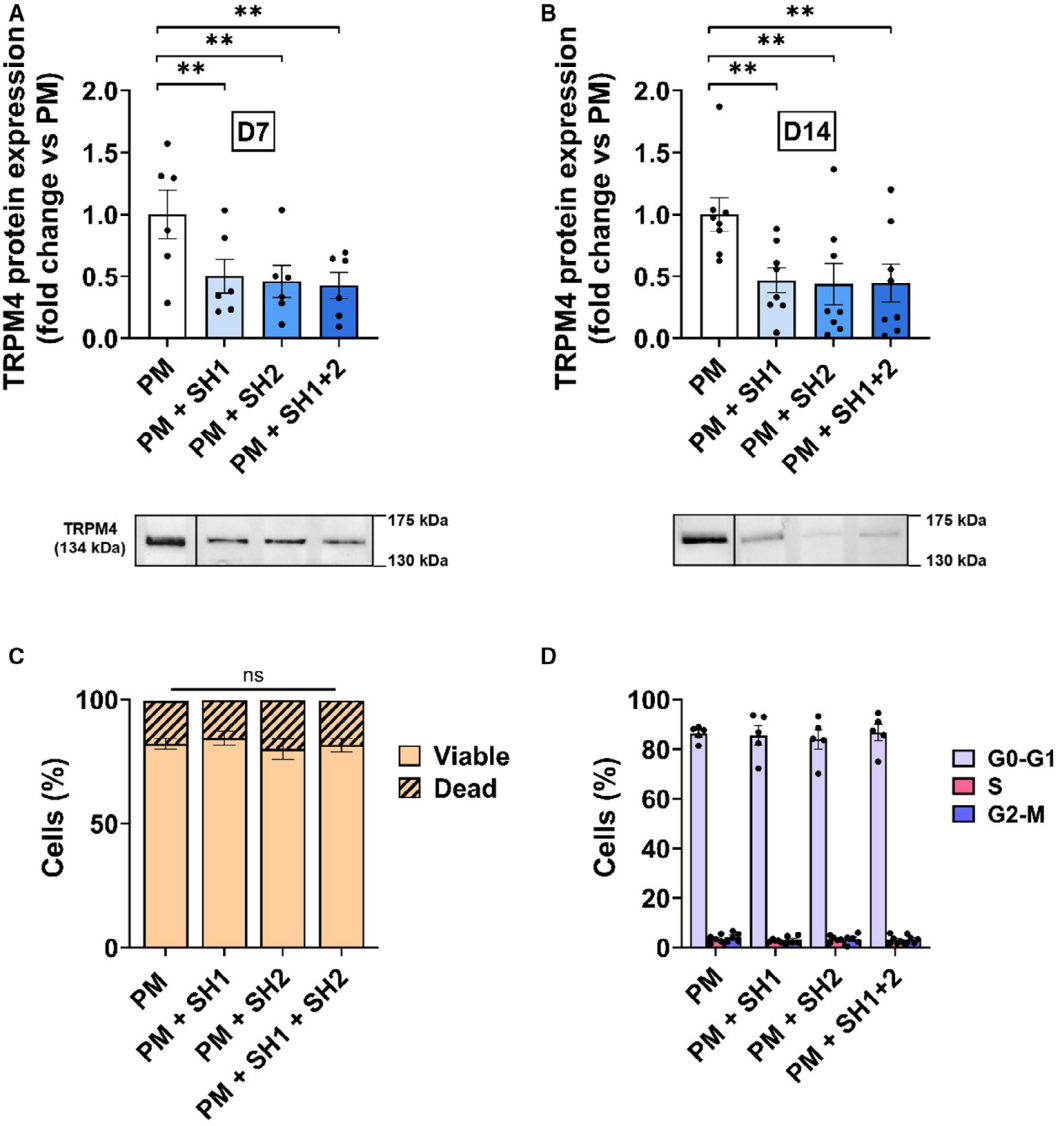
shRNA‐TRPM4 are functional and not toxic for hVICs. **A** and **B**, Quantification of TRPM4 protein expression in hVICs transduced with 1 ng/cm^2^ SH1, SH2 or SH1+2 after 7 days (N=6) (**A**) or 14 days (N=8) (**B)**. Representative western blots are provided below the histograms. Histograms are mean±SEM of fold change vs PM. **C**, hVIC viability evaluated by propidium iodide experiments in flow cytometry after 14 days of hVICs cultured in PM or PM+SH at 1 ng/cm^2^ (N=5). **D**, Cell cycle was determined by flow cytometry after 14 days of hVICs cultured in PM or PM+SH at 1 ng/cm^2^ (N=5). Analyzed with 1‐way ANOVA (**A**) with uncorrected Fisher's least significant difference multiple comparisons test, Friedman test (**B**) with Dunn's test and mixed‐effects analysis (**C**) with uncorrected Fisher's least significant difference test. **P<0.01, ns=nonsignificant. hVICs indicates human valvular interstitial cell; PM, pro‐calcifying media; shRNA, small hairpin RNA; and TRPM4, transient receptor potential melastatin 4.

**Figure 5:**
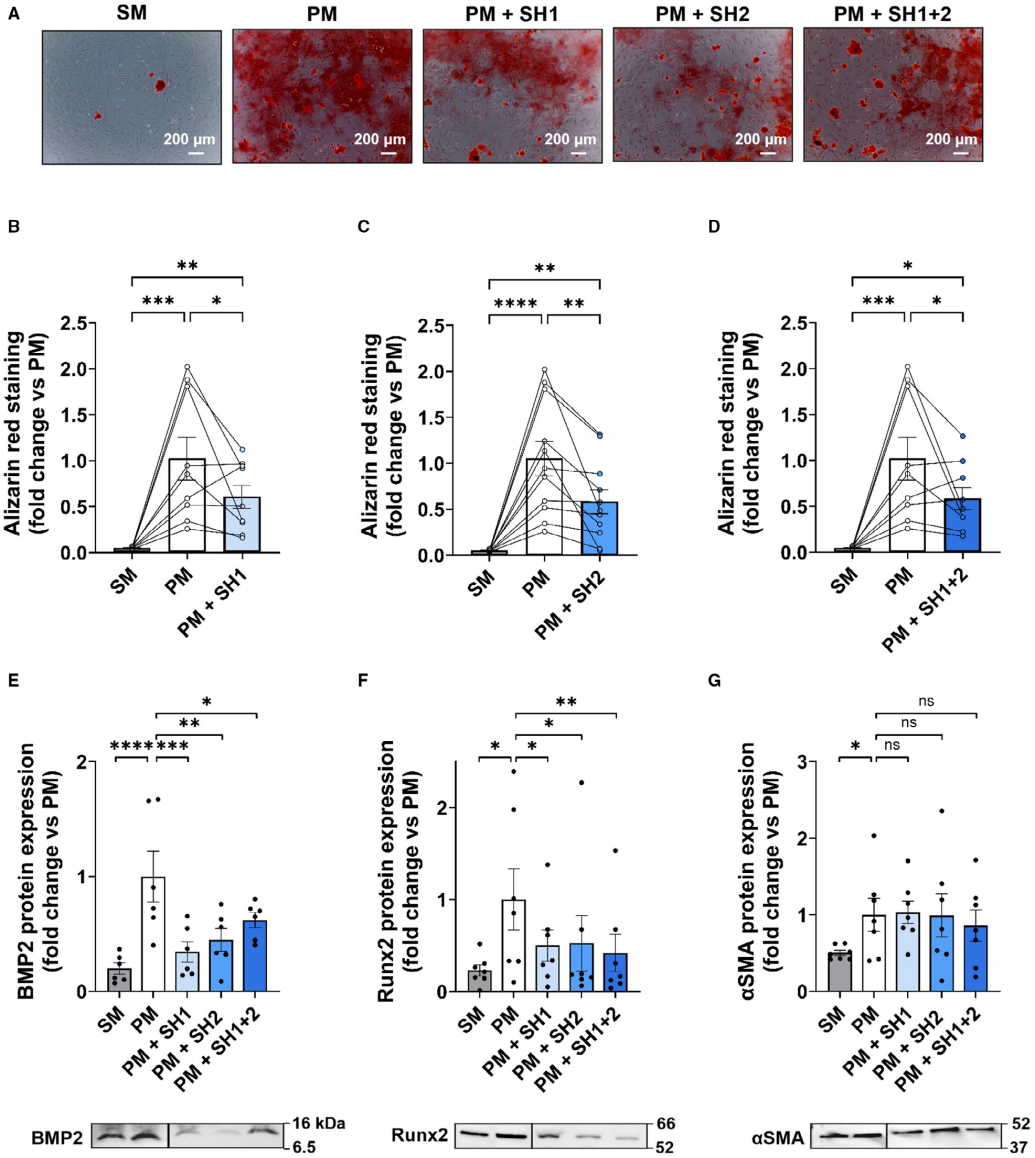
Repression of TRPM4 expression using shRNA‐TRPM4 reduces hVIC mineralization and osteogenic differentiation. **A**, Representative experiment showing calcium deposits measured by Alizarin red staining on hVIC after 14 days in culture. Cells were cultured in SM or PM with or without SH1, SH2, or SH1+2 at 1 ng/cm^2^. Scale bar=200 μm. **B–D**, Quantification of calcium deposits on hVICs after 14 days in culture in SM, PM, PM+SH1 (N=9, **B**), PM+SH2 (N=11, **C**) or PM+SH1+2 (N=9, **D**) as shown in **A**. Histograms are mean±SEM of fold change vs PM. Circles connected by lines represent data for each patient measured in the 3 conditions (average of duplicate). **E–G**, Quantification of BMP2 (N=6, **E**); Runx2 (N=7, **F**) and αSMA (N=7, **G**) after 14 days of hVIC culture in SM, PM, PM+SH1, PM+SH2 or PM+SH1+2 culture conditions. Analyzed with 1‐way ANOVA (**B–E**, **G**) with uncorrected Fisher's least significant difference multiple comparisons test and analyzed with Friedman test (**F**) with Dunn's multiple comparisons test. *P<0.05, **P<0.01, ***P<0.001, ****P<0.0001, ns=nonsignificant. BMP2 indicates bone morphogenetic protein 2; hVICs, human valvular interstitial cells; PM, pro‐calcifying media; Runx2, runt‐related transcription factor 2; shRNA, small hairpin RNA; SM, standard media; αSMA, α‐smooth muscle actin; and TRPM4, transient receptor potential melastatin 4.

In PM conditions, the effect of 1 ng/cm^2^ shRNA on TRPM4 protein expression was evaluated at 7 (N=6) and 14 days (N=8). SH1, SH2 or SH1+2 reduced TRPM4 expression by close to 40% (Figure 4A and 4B; Figure S5). Flow cytometry assays indicated that shRNA did not affect cell viability nor cell cycle at 14 days (N=5) (Figure 4C and 4D). Culture in PM condition with SH1 (N=9), SH2 (N=11) or SH1+2 (N=9) significantly reduced the mineralization level after 14 days compared with PM conditions alone (Figure 5). Increase in protein expression of osteogenic markers BMP2 and Runx2 induced by PM was prevented by shRNA after 7 and 14 days (N=6–7) (Figure S6; Figure 5). shRNA did not induce any significant variations in αSMA level (N=7–9) (Figure S6; Figure 5). Note that shScramble had no effect on mineralization, as well as TRPM4, BMP2, and Runx2 protein expression (N=4) (Figure S7).

To evaluate whether the effect of TRPM4 inhibition is dependent on valvular phenotype (bicuspid versus tricuspid), the data were reevaluated by comparing the results obtained from cells issued from bicuspid or tricuspid aortic valves. Indeed, it is known that bicuspid phenotype, which is found in around 1% of the general population, is a risk factor of valvular remodeling.^3^ PM condition induced a similar mineralization in both groups (Figure S8A) and TRPM4 inhibition by 9 PHE or repression by shRNA had similar effects on both groups (Figure S8B and S8C). Such similar results were also observed for the expression of osteogenic markers (Figure S9).

### Inhibition or Repression of TRPM4 Reduce the Activation of SMAD1/5 and NFAT Pathways

**Figure 6:**
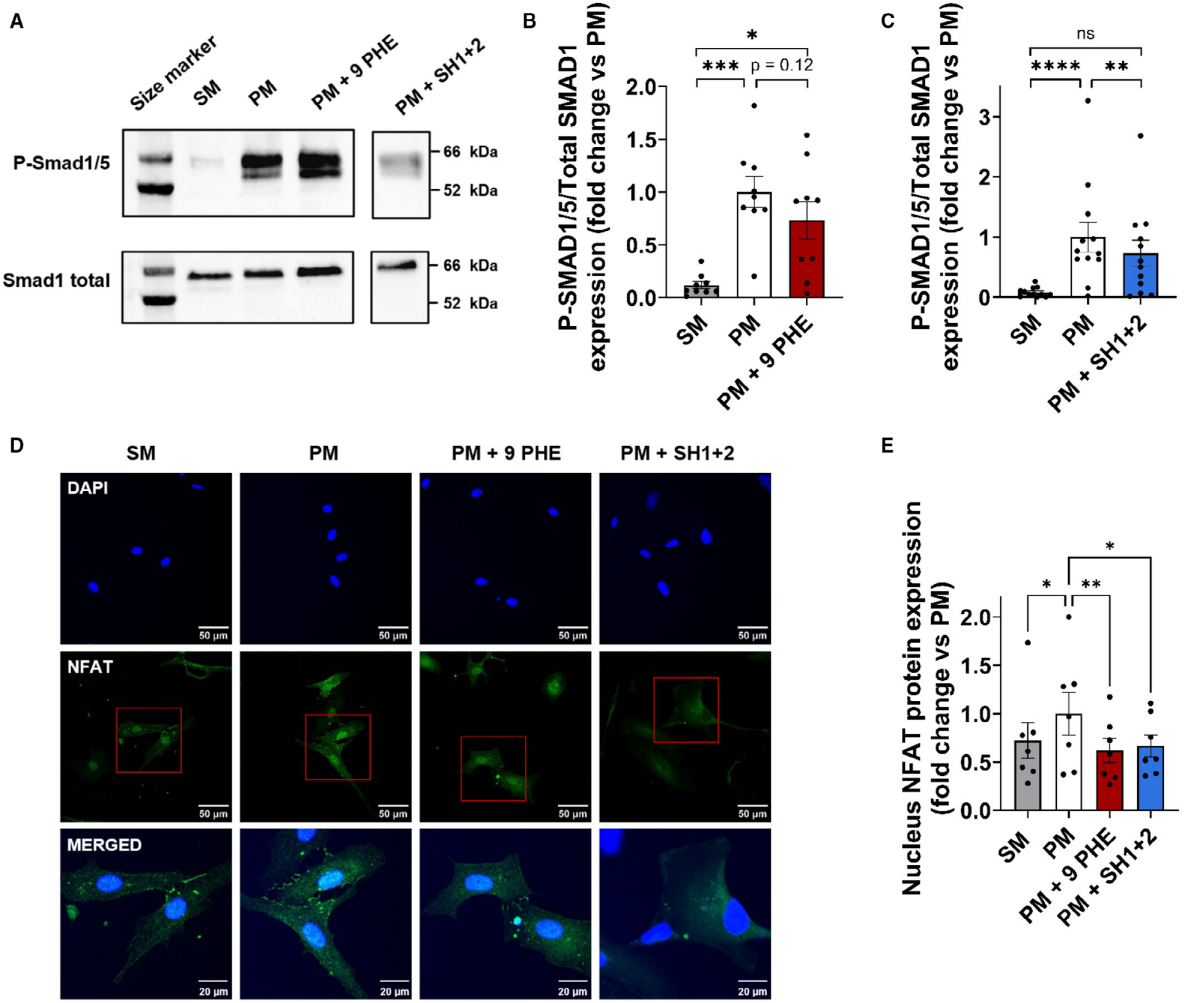
Inhibition and repression of TRPM4 reduces SMAD1/5 and NFAT pathways activation induced by PM. **A**, Western blots of P‐SMAD1/5 and SMAD1 total protein expression in hVICs after 14 days of culture in SM, PM, PM+9 PHE at 3.10^−6^ mol.L^−1^ or PM+SH1+2 at 1 ng/cm^2^. **B**, **C**, Quantification of P‐SMAD1/5 on SMAD1 total protein expression after 14 days of hVIC maintained in SM, PM, PM+9 PHE (N=9, **B**) or PM+SH1+2 (N=12, **C**) culture conditions. Histograms are mean±SEM of fold change vs PM. Analyzed with 1‐way ANOVA (**B**) with uncorrected Fisher's least significant difference multiple comparisons test and Friedman test (**C**) with Dunn's multiple comparisons test. **D**, NFAT protein expression (green) was observed by immunofluorescence in hVICs in SM, PM, PM+9 PHE or PM+SH1+2 (N=6) after 14 days of culture. Nuclei were labeled with DAPI (blue). Scale bar=50 μm. Upper line corresponds to the DAPI labeling and middle line corresponds to the NFAT labeling. The lower line corresponds to the merge (DAPI+NFAT) in the area indicated by a red square in the pictures of the middle line. **E**, NFAT protein expression was quantified by ImageJ software. Analyzed with 1‐way ANOVA with uncorrected Fisher's least significant difference multiple comparisons. *P<0.05, **P<0.01, ***P<0.001, ****P<0.0001, ns, non‐significant. 9 PHE indicates 9‐phenanthrol; hVICs, human valvular interstitial cells; NFAT, nuclear factor of activated T cells; PM, pro‐calcifying media; SM, standard media; and TRPM4, transient receptor potential melastatin 4.

Activation of SMAD1/5 pathway was evaluated by performing western blot of SMAD1/5 phosphorylation and total SMAD1 expression. PM induced an increase of 7.3‐fold of the ratio of phosphorylated SMAD1/5 over total SMAD1 compared with SM (Figure 6). 9 PHE (3.10^−6^ mol.L^−1^) or repression of TRPM4 by SH1+2 (N=12) partly prevented the increase in ratio induced by PM after 14 days of treatment of hVICs in culture (N=9) (Figure 6). The NFAT signaling, another pathway involved in osteogenic differentiation, was investigated. By confocal microscopy, the activated form of NFAT located in the nucleus was observed (N=6) (Figure 6D) and quantified (Figure 6E). PM induced a 39% increase of nuclear expression of NFAT in comparison with SM. Treatment by 9 PHE or shRNA‐TRPM4 reduced by 38% and 33%, respectively, the nuclear expression of NFAT compared with the cells cultured in PM alone.

## Discussion

Calcified aortic valve disease is a worrying disease worldwide. Indeed, a recent report indicates a 4.4‐fold increase in prevalence from 1990 to 2019 in the global population.^18^ Without pharmacological therapy to prevent or attenuate the disease, valvular replacement is the only option available. Identifying molecular actors is necessary to develop new therapeutic strategies. Here, we provide arguments supporting the involvement of TRPM4 in human VIC remodeling in vitro: (1) A typical TRPM4 current was recorded in hVICs; (2) pharmacological inhibition or protein repression of TRPM4 reduced hVIC remodeling; (3) TRPM4 contribution to hVIC remodeling involved BMP2, Runx2, and SMAD1/5 and NFAT signaling.

### Typical TRPM4 Channel Expressed in hVICs

A variety of ion channels are expressed on cardiac fibroblasts,^19^, ^20^ but only few were functionally reported in VICs. Three TRP isoforms (TRPC6, TRPM4, and TRPV4) were reported at the protein level on human VICs.^17^ Interestingly, their expression was higher in osteoblastic than in fibroblastic VIC in culture,^17^ in accordance with our results indicating a higher molecular and functional expression of TRPM4 in PM compared with SM conditions. Immunohistochemical labeling on aortic valve leaflet showed a stronger expression of TRPC3, TRPM4, and TRPV4 on VICs from patients with calcified aortic valves than from noncalcified ones^17^, ^21^ and their expression appeared in all layers of the leaflets. It indicates that enhanced expression observed in vitro under PM conditions is representative of the pathological conditions in vivo. Until now, no clear recordings of corresponding functional currents were reported. A functional expression of the TRPV4 channel was suspected on porcine VICs in culture, using a fluorescence resonance energy transfer–based Ca^2+^ sensor and a TRPV4 pharmacological activator, but corresponding ionic current was not reported.^22^ It was shown to participate in VIC transition to myofibroblasts.^10^, ^17^, ^22^

A genome‐wide association study detected an overexpression of the *CACNA1C* gene encoding for the α.1C subunit of the Ca_V_1.2 channel in calcified valves.^23^ Even if the valvular cell type expressing the Ca_V_1.2 was not identified, another study indicated that transfection with the *Ca*_*V*_ *1.2* gene of murine VICs in culture induced a transition of fibroblastic VICs to myofibroblasts.^24^ This is of importance regarding the contribution of TRPM4 to VIC remodeling since cell depolarization induced by TRPM4 activation might promote Ca_V_1.2 activation.

TREK‐1 and Kir 6.1 K^+^ channels were detected by western blot on hVICs. They might be the molecular determinants of a mechano‐sensitive K^+^‐selective current detected in 5% of excised inside‐out patches.^17^ In contrast, a linear current with a unitary conductance of 43 pS was detected in 95% of cell‐attached patches in the same study. This current was poorly characterized by the authors but hypothesized to be a nonselective cation current. If so, the molecular determinant of this current is unknown. It probably does not correspond to TRPV4 since the expected unitary conductance would have been from 80 to 100 pS.^25^ It also does not correspond to TRPM4 since the conductance is twice as high as expected. In addition, the authors introduced glibenclamide in their pipette solution, a compound known to inhibit the TRPM4 channel and thus preventing detection of this isoform.^26^ Finally, it might be consistent with the TRPC6 channel according to the unitary conductance and its protein expression in hVICs,^17^ but this remains to be evaluated.

In the present study, we report a current with a linear unitary conductance around 20 pS, an equal permeability for Na^+^ and K^+^, a higher activity in positive voltages, an activation by internal Ca^2+^, and a sensitivity to 9 PHE. These are the hallmark of the TRPM4 current.^14^, ^27^ This channel is widely expressed among human tissues^28^ and well characterized in cardiac cells including cardiomyocytes^29^ and atrial or ventricular fibroblasts.^16^, ^30^ This last cell type is of interest since it is close to VICs. TRPM4 is involved in the transition of human atrial fibroblasts to myofibroblasts in culture since this transition is reduced by 9 PHE.^16^ In addition, TRPM4 expression is higher in fibroblasts from patients with heart failure compared with control and in fibroblasts in culture after incubation by transforming growth factor β1.^30^ Such increased expression after transforming growth factor β1 treatment as well as participation in fibroblast to myofibroblast transition were also observed on mice cardiac fibroblasts.^16^, ^31^ This is of interest in the context of aortic stenosis since transforming growth factor β1 is known to induce VIC remodeling.^4^

A limitation of our study is that we focused on VICs, while TRPM4 might also be expressed in other valvular cell types such as valvular endothelial cells or immune infiltrating cells. Indeed, TRPM4 is expressed in human umbilical vein endothelial cells^32^ but not yet described in valvular endothelial cells. It is also expressed in immune cells including T lymphocytes^33^ and monocytes^34^ able to infiltrate the leaflets, or other immune cells derived from monocytes after infiltration such as macrophages or dendritic cells.^34^, ^35^ We focused on VICs, which are effectors in the processes of valvular fibrosis and calcification. Further studies remain to be conducted on other cell types to determine a possible role of TRPM4s in their contribution to valvular remodeling.

### TRPM4 in hVIC Remodeling

A probable mechanism by which TRPM4 can modulate VIC remodeling is an effect on Ca^2+^ homeostasis since this ion is involved in VIC remodeling^10^, ^24^ and TRPM4 modulates Ca^2+^ homeostasis in different cell types.^33^ This TRPM4‐dependent modulation can be done by acting on the electrochemical gradient for Ca^2+^ entry, TRPM4 inducing a depolarizing inward current that reduces this gradient. Conversely, depolarization induced by TRPM4 can activate voltage‐gated Ca^2+^ channels whose presence is attested on VICs.^23^, ^24^ Unfortunately, despite our efforts, we were not able to reliably measure the level of intracellular Ca^2+^ in our cells subjected to different culture conditions. This element will be important to evaluate in future studies in order to determine whether it is through the modulation of Ca^2+^ fluxes that TRPM4 influences cellular remodeling.

BMP2 is one of the main regulators of aortic valve mineralization. It activates a canonical pathway that includes SMADs and a noncanonical one in which extracellular signal‐regulated kinase activates the expression of Runx2.^9^ Since, in our experiments, TRPM4 inhibition leads to a reduction in phosphorylated SMAD1/5 and Runx2, we can hypothesize that it occurs through the upstream reduction of BMP2 expression, which was also observed. Moreover, we also observed that TRPM4 induced the nuclear translocation of NFAT. In human umbilical vein endothelial cells, it was shown that the activation of the transcriptional factor NFAT induced an increase of BMP2 expression.^36^ Therefore, we can suggest that TRPM4 can induced the nuclear translocation of NFAT, which conduct to the BMP2 protein expression that activate the SMAD1/5 pathway and therefore the expression of other osteogenic markers such as Runx2.

Interestingly, VIC treated by 9 PHE exhibit a reduction in their TRPM4 signal in western blot. It indicates that, in addition to the functional reduction in TRPM4 open probability, 9 PHE also produces a reduction in protein expression. Such observation was already reported in a model of traumatic brain injury in rat.^37^ It indicates that, in hVICs, TRPM4 might be implicated in signaling pathways regulating its own expression.

### Targeting TRPM4 in Calcified Aortic Valve

The actual therapy for aortic stenosis is mainly based on valve replacement due to the lack of approaches able to reduce the progression of valve remodeling. The discovery of TRPM4 as a contributor in this remodeling opens new possibilities but several obstacles remain to be overcome. Among these, we can note the need of TRPM4 inhibitors that can be used in vivo in humans. Very few TRPM4 pharmacological modulators are known, and their potential uses in vivo are poorly documented. Regarding 9 PHE, while this molecule was tested in a large variety of cells and tissues in vitro,^38^ to our knowledge, only 2 studies evaluated its effect when injected in vivo in mouse, and this was made with short time applications.^39^, ^40^ The same remark also applied for the TRPM4 inhibitor 4‐chloro‐2‐[2‐(2‐chloro‐phenoxy)‐acetylamino]‐benzoic acid.^39^, ^41^ An interesting avenue could come from the use of the antidiabetic glibenclamide, which inhibits TRPM4.^26^, ^42^ Clinical trials are underway to evaluate its effect in the context of nervous system damages in which TRPM4 is involved.^43^, ^44^ Another pitfall in the use of TRPM4 inhibitor in vivo might come from the large distribution of the channel within tissues^14^ and its contribution to a variety of vital physiological processes such as cardiac or breathing activities.^28^, ^45^ To induce an inhibition of TRPM4 in the aortic valve tissue without side effects, one might be able to address the molecules specifically in this tissue. A recent publication identified the protease‐activated receptor 2 as a protein upregulated on osteoblastic VICs.^46^ The authors used this protein as a target to specifically deliver drugs on VIC with magnetic nano‐cargoes. It thus paves the way for specific delivery of drugs to treat VIC remodeling. It shows that, even if the road is still long for a therapeutic treatment of aortic stenosis based on the modulation of TRPM4, this approach could be considered.

## Sources of Funding

This work was supported by the French Government, managed by the National Research Agency under the program “Investissements d'avenir” with the references ANR‐16‐RHUS‐0003 and ANR‐24‐CE14‐1977‐01. It was conducted as part of the “Fédération hospital‐Universitaire” CARNAVAL project (GSC G4). The “ministère de l'enseignement supérieur, de la recherche et de l'innovation” provided a PhD fellowship for Dr Aize, C. Kerevel, and Dr Bangando. The project benefits from a support from “GIP Cancéropôle Nord‐Ouest.”

## Disclosures

None.

## Supporting information

Data S1 STOP‐AS Investigators, Supplemental Methods
Tables S1–S4
Figures S1–S9
