# Supplementary material for: Contribution of the TRPM4 Channel to Osteogenic Differentiation of Human Aortic Valve Interstitial Cells
Source: J Am Heart Assoc. 2025 Apr 7;14(8):e038542. doi: 10.1161/JAHA.124.038542 (PMC12132880; doi:10.1161/JAHA.124.038542)
Supplement: Supplementary file 1 — Data S1 STOP‐AS Investigators, Supplemental Methods Tables S1–S4 Figures S1–S9 [file JAH3-14-e038542-s001.pdf]

Supplemental Material

STOP-AS INVESTIGATORS

first name	familly name	Affiliation
Hélène	Eltchaninoff	Univ Rouen Normandie, Inserm U1096, CHU Rouen, Department of Cardiology, F-76000 Rouen, France
Jérémy	Bellien	Univ Rouen Normandie, Inserm U1096, CHU Rouen, Department of Pharmacology, F-76000 Rouen, France
Benjamin	Bertrand	Cardiawave, Paris, France
Farzin	Beygui	Service de Cardiologie, ACTION Study Group, Centre Hospitalier Universitaire (CHU) de Caen Normandie, Normandie Univ, INSERM UMRS 1237, GIP
Delphine	Béziau-Gasnier	CHU Rouen, Department of Cardiology, F-76000 Rouen, France
Ebba	Brakenhielm	Univ Rouen Normandie, Inserm U1096, F-76000 Rouen, France
Giuseppina	Caligiuri	Université de Paris, Paris, France; Laboratory for Vascular Translational Science, INSERM U1148, Paris, France
Karine	Chevreul	Inserm, ECEVE, Paris, France; Université Paris Cité, Paris, France; URC Eco Ile de France, Assistance Publique-Hôpitaux de Paris, Hôtel Dieu, Paris, France
Frédérique	Debroucker	Medtronic, Boulogne-Billancourt, France
Eric	Durand	Univ Rouen Normandie, Inserm U1096, CHU Rouen, Department of Cardiology, F-76000 Rouen, France
Christophe	Fraschini	Supersonic Imagine, Aix-en-Provence, France
Martine	Gilard	Université de Bretagne Occidentale, Brest, France
Bernard	Iung	Department of Cardiology, Bichat Hospital, AP-HP, 75018 Paris, France; Inserm U1148, 75018 Paris, France; University of Paris Cité, 75018 Paris, France
Said	Kamel	UR UPJV 7517, MP3CV, CURS, Université de Picardie Jules Verne, Amiens, France; Department of Biochemistry, Amiens University Hospital, Amiens, France
Jamila	Laschet	Université de Paris, Paris, France; Laboratory for Vascular Translational Science, INSERM U1148, Paris, France
Alain	Manrique	Normandie Université, UR 4650, Physiopathologie et Stratégies d'Imagerie du Remodelage Cardiovasculaire, GIP Cyceron, CHU de Caen, UNICAEN, Campus Jules Horowitz, BP 5229, 14074 Caen, France
Emmanuel	Messas	Vascular medicine department, Georges Pompidou European hospital, APHP, Paris University Paris France; INSERM U970 PARCC, Université Paris Cité, Paris, France
David	Messika-Zeitoun	Department of Cardiology, University of Ottawa Heart Institute, Ottawa, ON,
Florence	Pinet	UniversityLille, Inserm, CHU Lille, Institut Pasteur de Lille, U1167 - RID-AGE - Facteurs de risque et déterminants moléculaires des maladies liées au vieillissement, 59000 Lille, France
Vincent	Richard	Univ Rouen Normandie, Inserm U1096, CHU Rouen, Department of Pharmacology, F-76000 Rouen, France
Eric	Saloux	Department of Cardiology, Normandie Univ, UNICAEN, CHU de Caen Normandie, EA4650 (SEILIRM), 14000, Caen, France
Martin	Thoenes	Edwards Lifesciences, Nyon, Switzerland
Christophe	Tribouilloy	Department of Cardiology, Amiens University Hospital, 80054 Amiens, France; UR UPJV 7517, Jules-Verne University of Picardie, 80054 Amiens,
Claire	Vézier	CHU Rouen, Department of Cardiology, F-76000 Rouen, France

Data S1.

Supplemental Methods

Methods for quantitative reverse transcriptase polymerase chain reaction RT-PCR :

Total RNA was extracted from hVIC by GenElute™ Total RNA (Sigma Aldrich, USA) according to the manufacturer instructions. Total RNA was quantified by spectrophotometry (Nanodrop Technologies, Wilmington, USA). cDNA was synthesized from 1 µg of total RNA by using The High Capacity cDNA Reverse Transcription Kit (Thermofisher Scientific, Lithuania) with the following cycle conditions : 25°C (10 min), 37°C (120 min), 85°C (5 min). Primers (Eurogentec) for the amplification were designed with Primer3Plus (https://www.bioinformatics.nl/cgi-bin/primer3plus/primer3plus.cgi) and listed in Table S3. cDNA was amplified with the following cycles : 95°C (5 min); [94°C (30 s), 60°C (30 s), 72°C (90 s)]x35; 72°C (5 min). Presence of cDNA was revealed on 2% agarose gel was revealed with imageQuant LAS 4000.

Methods for qPCR :

The PCR amplification was carried out with 1 µg of cDNA, primers at 10 µM and iQ™ SYBR Green Supermix (Bio-Rad, France). Thermal cycles were performed in a Bio-Rad® CFX96™ according to supplier recommendations : 95°C (3 min); [95°C (2 s), 60°C (20 s)]x39; 70°C (30 s). Each plate contains triplicate for each culture conditions. Cq values were obtained from the Bio-Rad CFX manager Software. Relative gene expression values were corrected with each efficiency (E) calculated with the following equation : $E = 10^{-(1/slope)}$.

Methods for cytometry :

Cell viability and cycle was evaluated by flow cytometry by propidium iodide experiments. hVIC were cultured for 14 days under standard (SM) or pro-calcifying (PM) media in the presence or absence of 9-phenanthrol or shRNA. Supernatant was collected during all this time

in culture and stored at 4°C. At 14 days, hVIC were washed with PBS, resuspended in PBS after trypsin treatment and incubated with 50 µg.mL^{-1} propidium iodide, 600 µg.mL^{-1} RNase in PBS for 10 min. Fluorescence, which depends on dead cells, was measured at 617 nm by Cytoflex-S (Beckman Coulter, USA). For cell cycle after 14 days of culture, cells were washed with PBS and resuspended in 70% of ethanol after trypsin treatment and conserved at -20°C. At the time of analysis, cells were centrifuged, incubated in PBS during 30 min at 37°C and placed in a mixture of 50 µg.mL^{-1} propidium iodide, 600 µg.mL^{-1} RNase in PBS during 15 min in obscurity at room temperature. Cell cycle was analyzed by Cytoflex-S (Beckman Coulter, USA).

Table S1: Data for patients with calcified valves

Parameters	Value (N = 74)	SEM
**Age (years)**	65.26	± 1.28
**Sex (%)**	74% M (55) / 26% F (19)	
**Tricuspid/ Bicuspid (%)**	60% T (44) / 40% B (30)	
**LVEF (%)**	59.49	± 1.75
**ΔPmean (mmHg)**	46.11	± 1.83
**Aortic valve jet Vmax ($m.s^{-1}$)**	4.10	± 0.13
**AVS (cm^2/m^2)**	0.50	± 0.02

LVEF : Left ventricular ejection fraction; ΔPmean : mean transaortic pressure gradient; Vmax : maximal velocity; AVS : aortic valve surface; M : male; F : female; T : tricuspid; B : bicuspid.

Table S2: Data for patients with non-calcified valves

Parameters	Value (N = 7)	SEM
**Age (years)**	58.00	± 5.04
**Sex (%)**	86% M (6) / 14% F (1)	
**Tricuspid/ Bicuspid (%)**	43% T (3) / 57% B (4)	
**LVEF (%)**	53.95	± 3.27
**Aortic valve jet Vmax ($m.s^{-1}$)**	1.67	± 0.22

LVEF : Left ventricular ejection fraction; Vmax : maximal velocity; M : male; F : female; T : tricuspid; B : bicuspid.

Table S3: PCR primers (5'→ 3')

Gene	Primers
**TRPM4**	F : ACCTAGTGGCTCTCACCTG
	R : AGTCGATGCAGAGGACAGT
**PECAM-1**	F : GACGTCGAATACCAGTGTGT
	R : CACCACCTTACTTGACAGGA
**VE-cadherin**	F : TACCAGCCCAAAGTGTGT
	R : CGTGGTGTTATGTCCTTGTC

Table S4: List of antibodies used for immunofluorescence (IF) and Western Blot

Methods	Antibody	Primary/Secondary	Dilution	Reference
IF	Rabbit polyclonal anti-TRPM4	Primary	1 : 400	Alomone Labs ACC-044
	Mouse monoclonal anti-αSMA	Primary	1 : 2000	Antibodies A36319
	Rabbit polyclonal anti-NFAT1	Primary	1 : 100	Cell signaling 4389
	Fluorescein (FITC) AffiniPure Donkey Anti-Rabbit IgG (H+L)	Secondary	1 : 800	Jackson ImmunoResearch 711-095-152
	Cy™3 AffiniPure Donkey Anti-Mouse IgG (H+L)	Secondary	1 : 800	Jackson ImmunoResearch 715-165-150
Western Blot	Anti-TRPM4	Primary	1 : 5000	Abcam ab123936
	Anti-Runx2	Primary	1 : 300	Santa Cruz sc-390715
	Anti-BMP2	Primary	1 : 500	RnDsystems MAB3551
	Anti-P-Smad1/5	Primary	1 : 1000	Cell Signaling 9516
	Anti-Smad1	Primary	1 : 1000	Cell Signaling 6944
	Anti-mouse IgG, HRP-linked Antibody	Secondary	1 : 5000	Cell Signaling 7076
	Anti-rabbit IgG, HRP-linked Antibody	Secondary	1 : 5000	Cell Signaling 7074

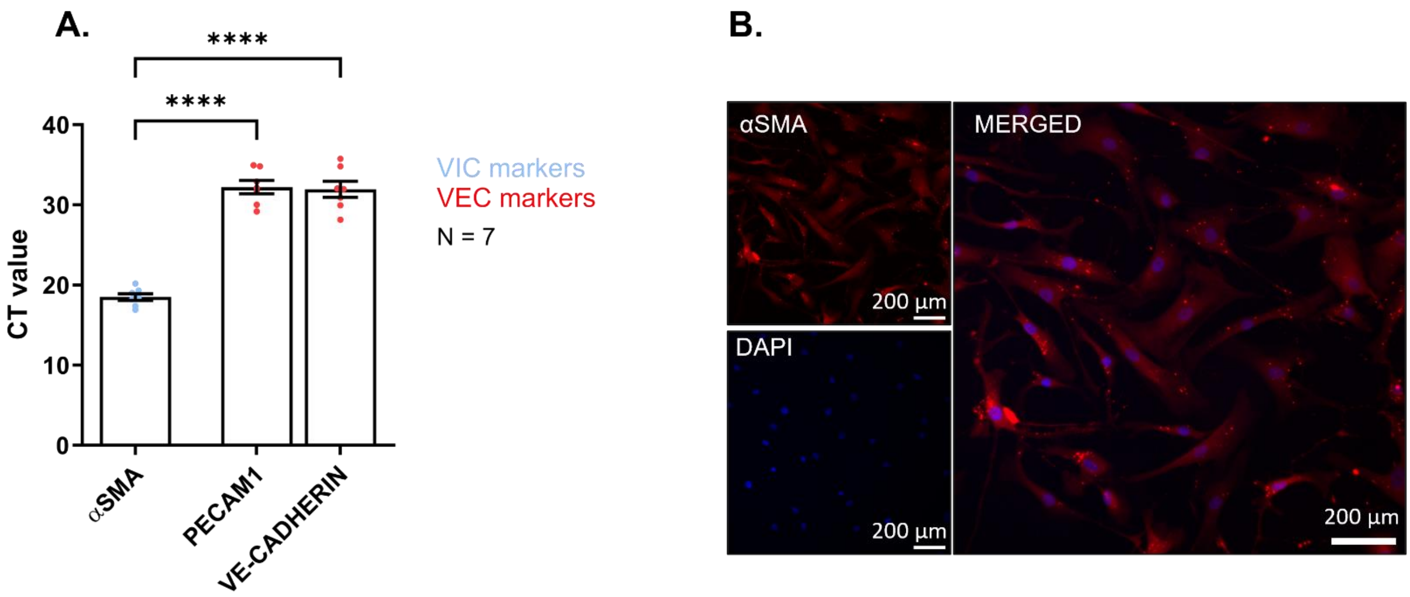

Figure S1: characterization of hVIC by qPCR and immunofluorescence.

A : Detection of hVIC marker α-smooth muscle actin (αSMA) and hVEC markers platelet endothelial cell adhesion molecule 1 (PECAM-1) and vascular endothelial-cadherin (VE-cadherin) by RT-qPCR (N = 7). Analyzed with One-Way Anova with Uncorrected Fisher's LSD multiple comparisons. **** $p < 0.0001$. **B :** Representative experiment of αSMA expression in hVIC detected by immunofluorescence (N = 6).

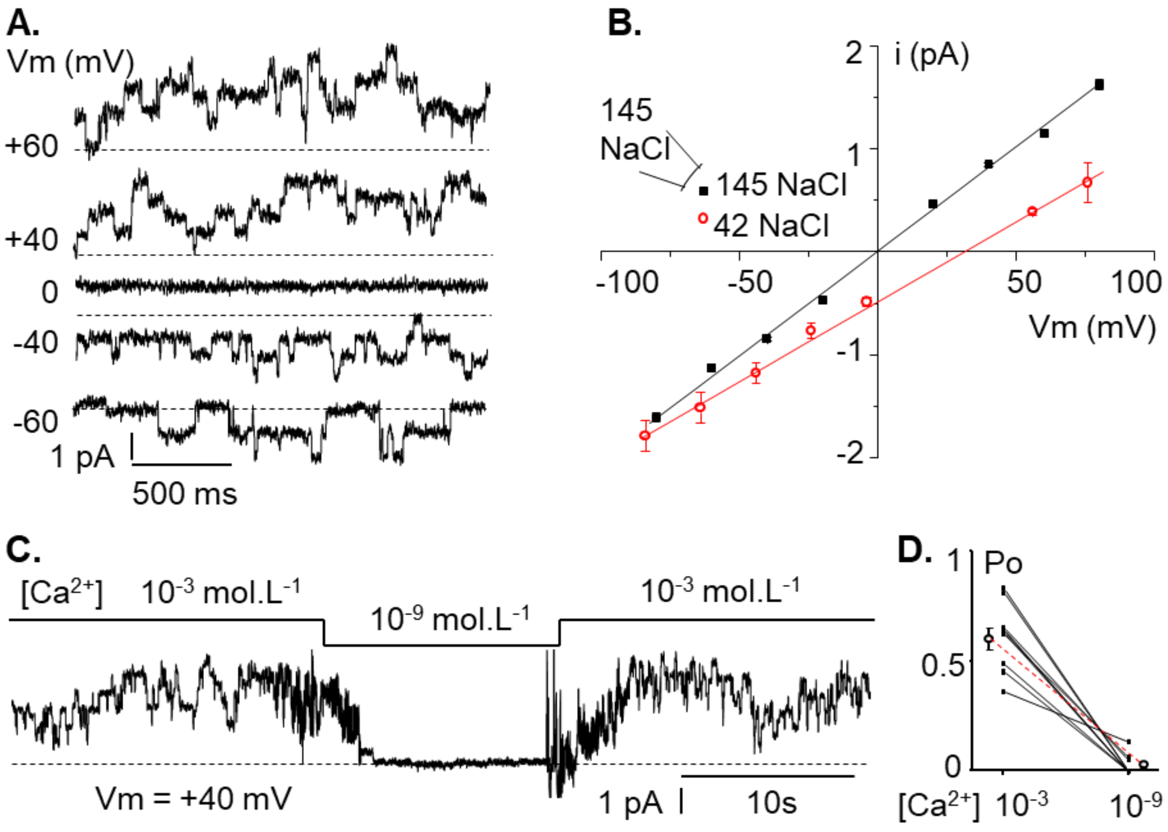

Figure S2: TRPM4 is functional on VIC after 14 days in PM conditions.

A : Single channel tracings recorded at various membrane potentials (V_m) from an inside-out patch from hVIC maintained from 14 days in PM conditions. Pipette and bath contained 145 mmol.L^{-1} NaCl standard solution ($CaCl_2$ = 10^{-3} mol.L^{-1}). Dashed lines indicate current level when all channels are closed. Note that channel activity is higher in the positive voltages. **B :** Black squares: Current voltage relationship (i/V_m) with 145 mmol.L^{-1} NaCl standard solution on both side of the membrane. Data points were fitted to a linear regression indicating a linear conductance g = 20.5 ± 0.4 pS (n = 22 , N = 3). Red open circles: current voltage relationship with the 145 mmol.L^{-1} NaCl standard solution in the pipette and 42 mmol.L^{-1} NaCl solution in the bath, revealing the cationic permeability of the channel (n = 6 , N = 2). **C :** Typical recording of an inside-out patch in the symmetrical 145 mmol.L^{-1} NaCl conditions and with a concentration of $CaCl_2$ at the inside of the membrane which was switched from 10^{-3} to 10^{-9} mol.L^{-1}, as indicated (V_m = +40 mV). **D :** P_o determined at 10^{-3} and 10^{-9} mol.L^{-1} $CaCl_2$ for 10 experiments similar to that shown in C. Black squares linked by plain lines correspond to single experiments. Open circles linked by red dotted line correspond to the mean ± SEM (n = 10 , N = 2).

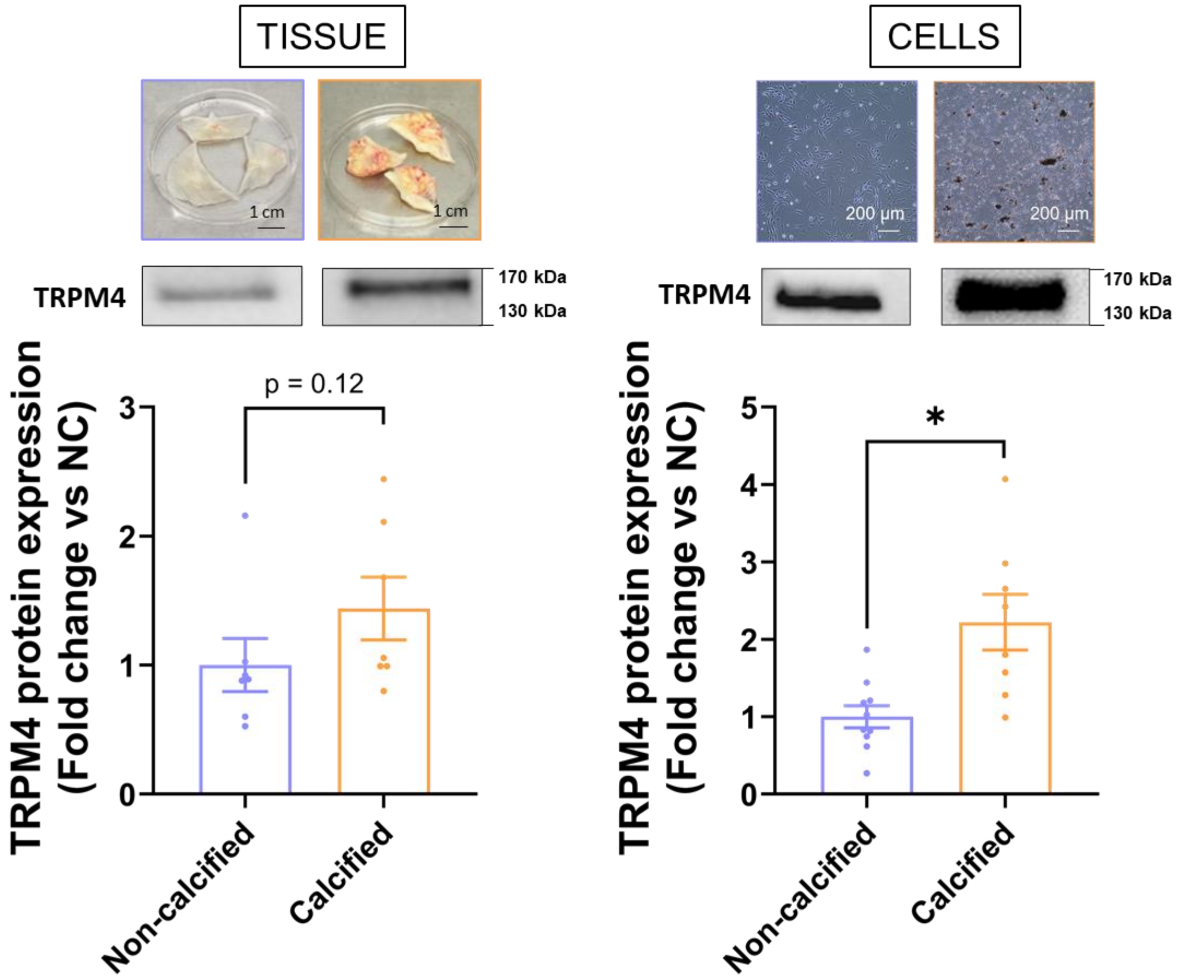

Figure S3: Comparison of TRPM4 protein expression between non-calcified and calcified valves.

Quantification of TRPM4 protein expression of Western Blots from non-calcified (N = 7) and calcified (N = 7) valves (left panel) and from hVIC from non-calcified (N = 10) and calcified (N = 8) valves (right panel). Upper pictures show representative valve leaflets and cell culture from each type.

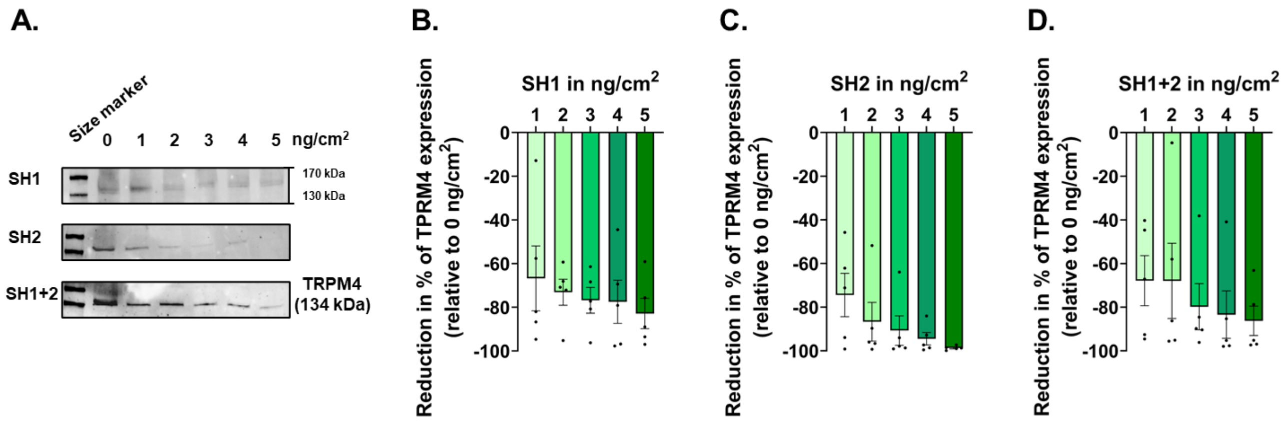

Figure S4: shRNA-TRPM4 are efficient in hVIC.

A : Representative Western Blots of TRPM4 protein expression in hVIC transduced for 14 days with shRNA-TRPM4 (SH1, SH2 or SH1+2) at several concentrations (0 to 5 ng/cm^2). **B-D :** TRPM4 expression in hVIC transduced with shRNA-TRPM4 was compared for each patient (N = 5) to the expression on control condition (without shRNA) after 14 days as indicated in A. Cells were transduced with SH1 (B), SH2 (C) or SH1+2 (D). Histograms are mean ± SEM of variation in TRPM4 expression compared to 0 ng/cm^2.

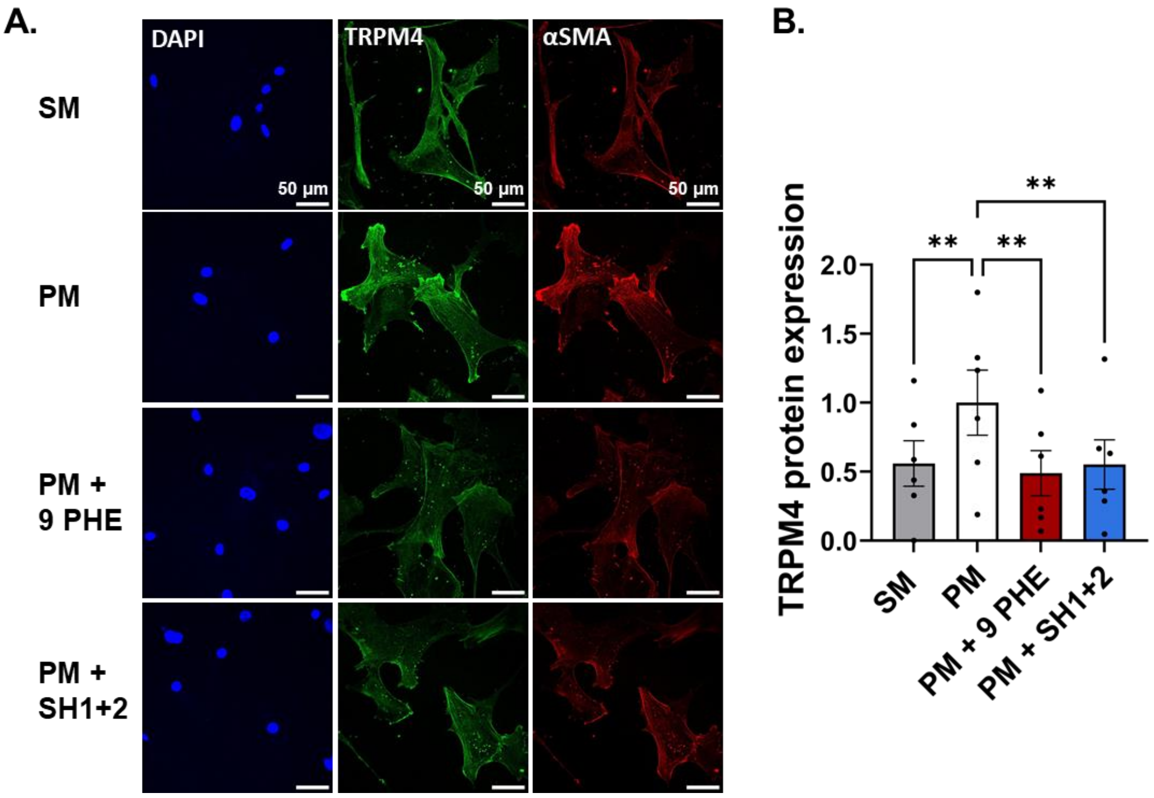

Figure S5: localization of TRPM4 expression in hVIC by immunofluorescence.

A : TRPM4 protein expression (green) was observed in hVIC in SM, PM, PM + 9 PHE or PM + SH1+2 (N = 6) after 14 days of culture by immunofluorescence. Nuclei were labeled with DAPI (blue) and total cell area was detected by αSMA labeling (red). Scale bar = 50 µm. **B :** TRPM4 protein expression was quantified by ImageJ software. Analyzed with One-Way Anova with Uncorrected Fisher's LSD multiple comparisons. ** $p < 0.01$.

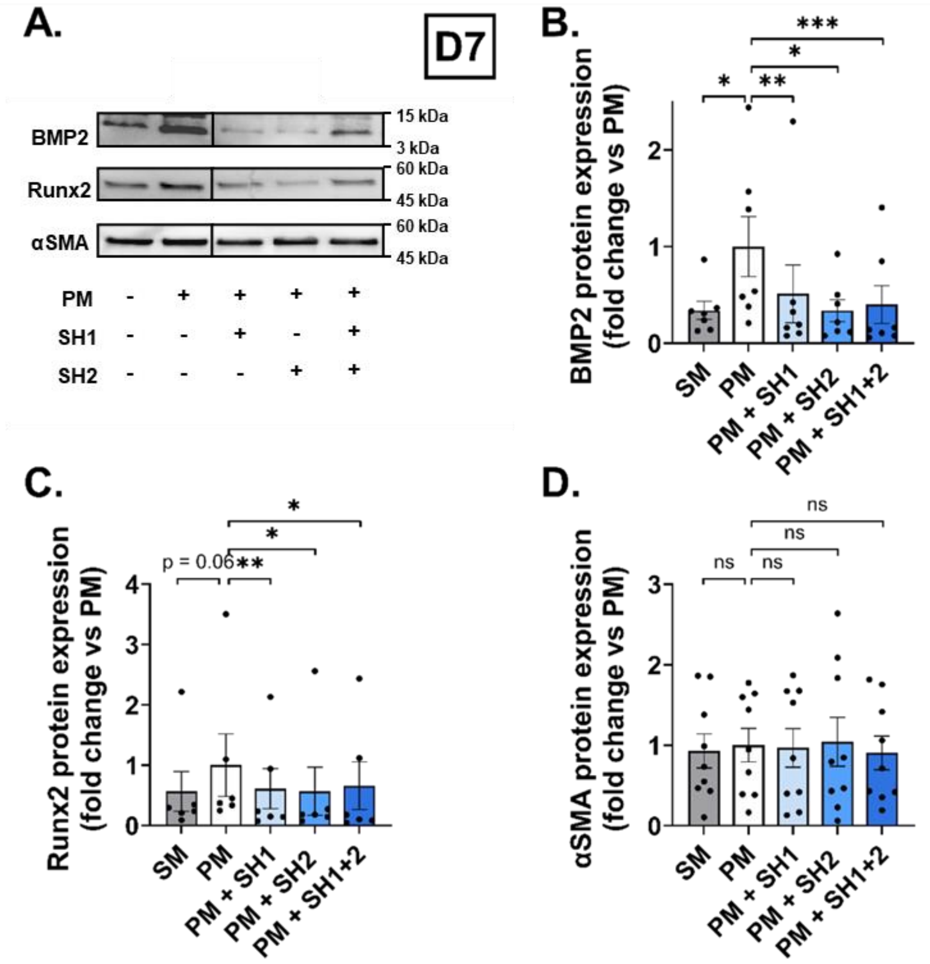

Figure S6: Reduction of osteogenic markers after hVIC transduction with shRNA-TRPM4.

A : Representative Western Blots of BMP2, Runx2 and α-SMA protein expression in hVIC after 7 days of culture. As indicated above panels, cells were cultured in SM conditions or in PM conditions without or with SH1, SH2 and SH1+2 at 1 ng/cm^2. **B-D :** Quantification of BMP2 (N = 7) (B), Runx2 (N = 6) (C) and α-SMA (N = 9) (D) after 7 days of hVIC cultured in SM, PM or PM + SH1, PM + SH2 or PM + SH1+2 culture conditions. Analyzed with Friedman test (B, C) with Dunn's multiple comparisons test and analyzed with One-Way Anova (D) with Uncorrected Fisher's LSD multiple comparisons test. * p <0.05, ** p <0.01, *** p < 0.001, ns = non-significant. Data are mean of fold change vs PM ± SEM.

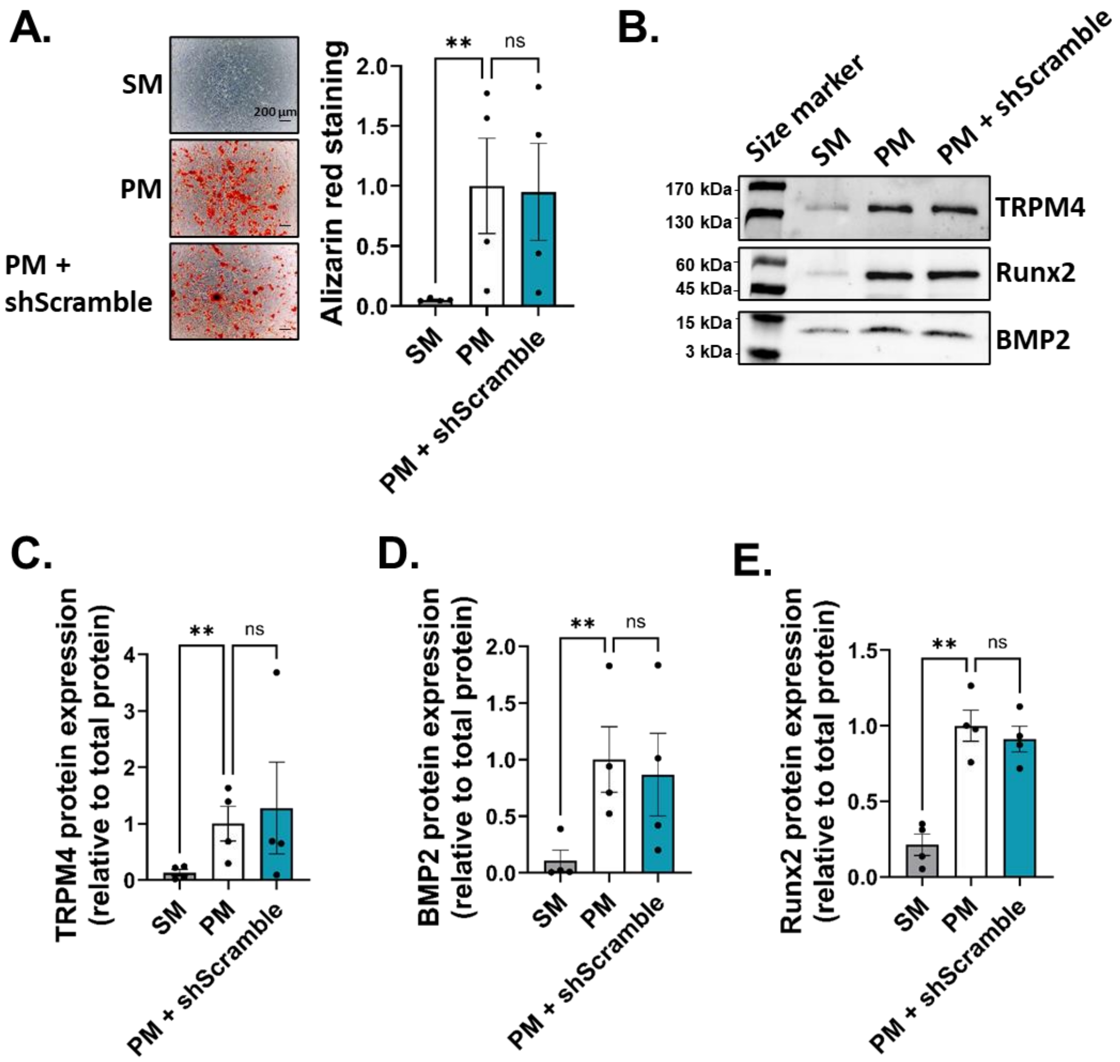

Figure S7: Effect of shRNA-Scramble on the mineralization and expression of osteogenic markers and TRPM4.

A : Mineralization was quantified after 14 days of culture in SM, PM or PM + shScramble (N=5). **B :** Representative Western Blots of TRPM4, Runx2 and BMP2 protein expression in hVIC after 14 days of culture. **C-E :** Quantification of TRPM4 (C), BMP2 (D), Runx2 (E) protein expression after 14 days of culture in SM, PM or PM + shScramble (N = 4). Analyzed with Friedman test with Dunn's multiple comparisons test. ** $p < 0.01$, ns = non-significant. Data are mean of fold change vs PM ± SEM.

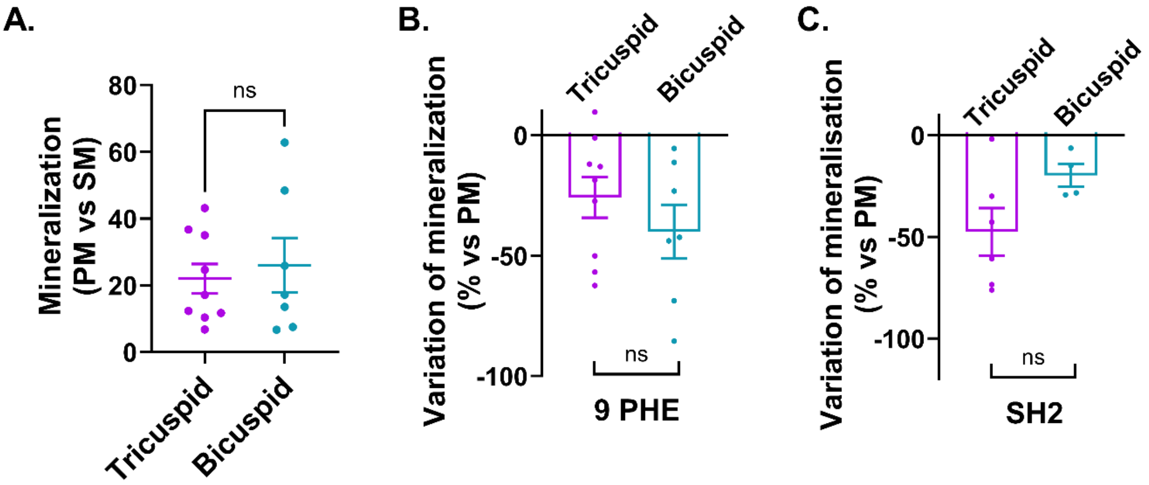

Figure S8: hVIC mineralization development depending on aortic valve phenotype (tricuspids or bicuspids).

A : Mineralization was quantifying after 14 days of culture in SM or PM on hVIC issued from tricuspids (N = 9) or bicuspids valves (N = 7). Data are express as a ratio of Alizarin red staining level in PM versus SM. **B-C :** Effect of 9-phenanthrol (B) or shRNA (SH2) on hVIC mineralization in PM depending on valve phenotype. Valve phenotype did not influence any of these parameters. Analyzed with unpaired t-test. ns = non-significant. Data are mean ± SEM.

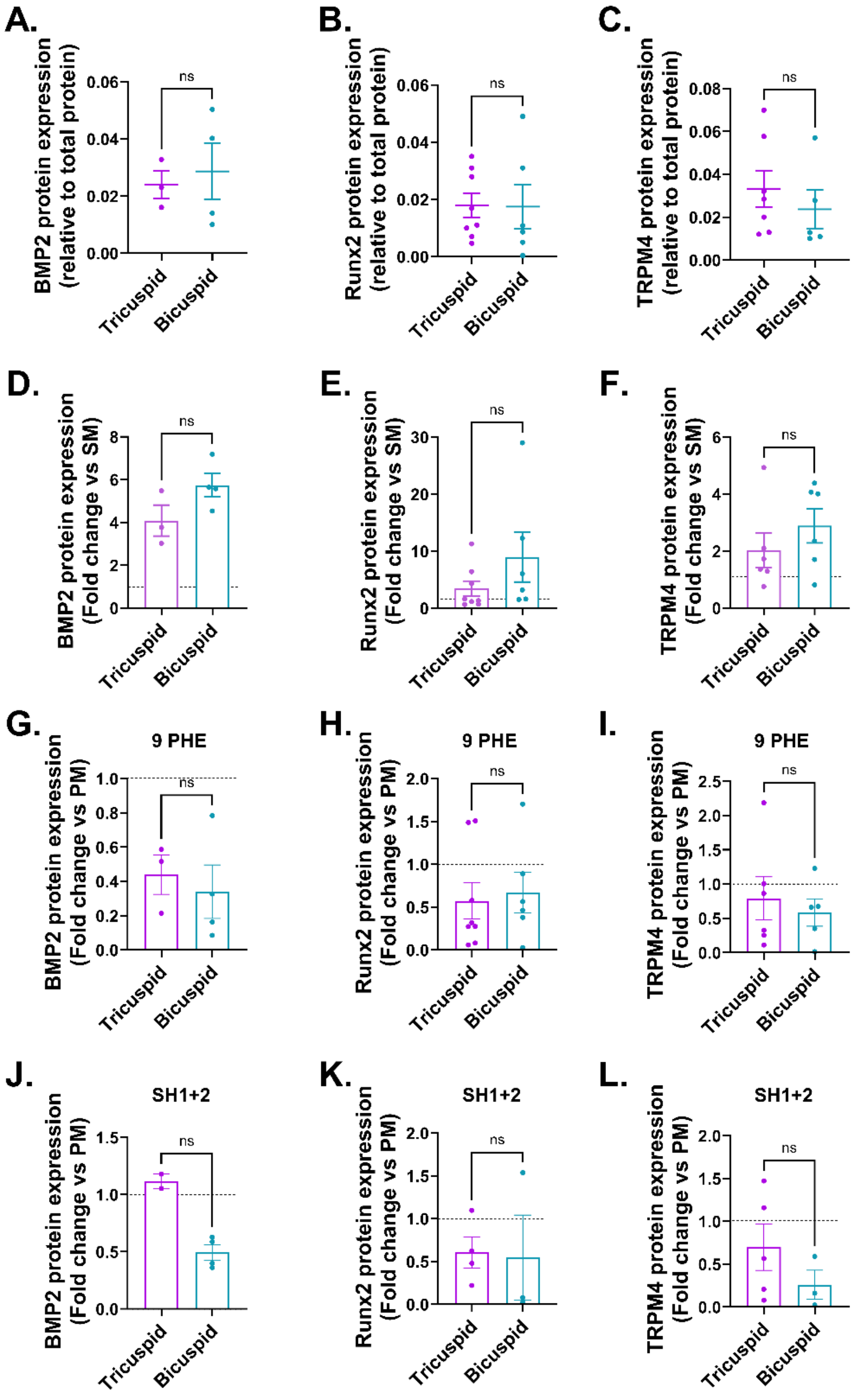

Figure S9: Osteogenic makers and TRPM4 protein expression in hVIC depending on aortic valve phenotype (tricuspid or bicuspid).

A-C : The expression of BMP2, Runx2, and TRPM4 was evaluated in hVIC issued from tricuspid and bicuspid valves after 14 days of culture in SM. **D-F :** The effect of PM compared to SM on the expression of the different markers mentioned above was studied on hVIC issued from tricuspid and bicuspid valves after 14 days of culture. **G-L :** The effect of 9-phenanthrol (G-I) or shRNA (J-L) treatment versus PM was investigated on hVIC derived from tricuspid

and bicuspid valves after 14 days of culture. Valve phenotype did not influence any of these parameters. Analyzed with unpaired t-test (A-C, I) and Mann Whitney test (D-H, J-L). ns = non-significant. Data are mean ± SEM.
